# Incidence of pests and viral disease on pepino (*Solanum muricatum* Ait.) in Kanagawa Prefecture, Japan

**DOI:** 10.3897/BDJ.5.e14879

**Published:** 2017-08-22

**Authors:** Ok-Kyung Kim, Tadashi Ishikawa, Yoshihiro Yamada, Takuma Sato, Hirosuke Shinohara, Ken Takahata

**Affiliations:** 1 Laboratory of Plant Pathology, Faculty of Agriculture, Tokyo University of Agriculture, Atsugi-shi, Kanagawa, Japan; 2 Laboratory of Entomology, Faculty of Agriculture, Tokyo University of Agriculture, Atsugi-shi, Kanagawa, Japan; 3 Laboratory of Vegetables, Faculty of Agriculture, Tokyo University of Agriculture, Atsugi-shi, Kanagawa, Japan

**Keywords:** insects, mites, virus vector, virus, sweet cucumber

## Abstract

**Background:**

The solanaceous fruit crop pepino (*Solanum muricatum* Ait.), originating in the Andes, is grown commercially in South American countries and New Zealand. In these areas, pests and diseases of pepino have been identified well; however, to date, these have seldom been investigated in detail in Japan. Herein, we attempt to reconstruct an agricultural production system for commercial pepino crops in Japan, and evaluate the incidence of pests and viral diseases on pepino. The findings of this study will facilitate in developing a better crop system for the commercial cultivation of healthy pepino fruits.

**New information:**

A total of 11 species, comprising nine insects and two mites, were recognized as pests of pepino plants in our experimental fields in Kanagawa Prefecture, central Honshu, Japan. Of these pest species, the two-spotted spider mite *Tetranychus urticae* Koch, 1836 and the cotton aphid *Aphis gossypii* Glover, 1877, were remarkably abundant than the other pest species. Eventually, 13 species, including two previously recorded, are currently recognized as the pests of pepino in Japan. With regard to viruses, we tested two species *Alfalfa mosaic virus* (AMV) and *Cucumber mosaic virus* (CMV), as well as three genera *Carlavirus*, *Potexvirus*, and *Potyvirus*. No virus was detected in symptomatic pepino leaves collected in our experimental fields. This is a first report on the identification of pests on pepino plants in Kanagawa Prefecture, Japan and elucidates the relationship between currently occurring pests of pepino plants and potential viral pathogens that they can transmit.

## Introduction

Pepino, the Spanish name for sweet cucumber, (*Solanum muricatum* Ait.), is a solanaceous plant cultivated as a fruit crop. It originated in the Andes, became popular in several countries and regions of South America (Heiser 1964), and then it was introduced to Central America and New Zealand. In Japan, cultivation of pepino began in 1984 based on pepino fruits imported from New Zealand in 1983 (Sakata 2011). Since then, pepino caused a major boom and its cultivation rapidly spread throughout Japan within a few years. However, the production gradually declined prior to 1990 due to the low soluble solids content (Brix) in the Japanese pepino fruits (less than 8°Brix) (Sakata 2011). Currently, farmers in Japan do not grow pepino, except for people with gardening as a hobby, who cultivate pepino.

In 2016, our research team began a project for regional development “Launching of Nodai-branded Pepino Crop” conducted by Faculty of Agriculture, Tokyo University of Agriculture (TUA; Nodai is a Japanese abbreviated name of the university). The main purpose of this project was to produce high quality and flavorsome pepino fruits with sufficient soluble solids content. As a recent achievement of this project, Takahata (2017) succeeded in increasing the soluble solids content of pepino fruits by using a washer ring (metal washer) at the bottom of the stem. This technique strongly contributes to improving the quality and flavor of pepino fruits.

To date, at least 24 species of insect and mite pests on pepino (Larraín 2002) and one virus infected to pepino (Jones et al. 1980) have been recorded in the native range of pepino, the Andes. In contrast, in regions where pepino was introduced other than Japan, such as New Zealand, China and Turkey, there are only a few records on the pests (two species: Galbreath and Clearwater 1983, Akyazi 2012) and viruses (two species: Thomas et al. 1980, Abouelnasr et al. 2014) in the academic literature. In Japan, little has been known in detail about the pests and viruses of pepino, except that inadequately identified pests such as spider mites and aphids damage to pepino.

It is important to establish solid pest control in its commercial cultivation to produce high quality and stable pepinos. Unfortunately, however, no pesticides applicable to pepino plants have been registered in Japan; this could be attributed to the few detailed studies on pests and diseases of pepino. Therefore, our research team has tried to comprehensively elucidate the pests and viral diseases of pepino in this project in order to contribute to the accumulation of basic knowledge toward the establishment of its pest control. This paper documents the results of our field survey on pests and diseases of pepino in Kanagawa Prefecture, central Japan.

## Materials and methods

### Study sites

**Figure 1.**
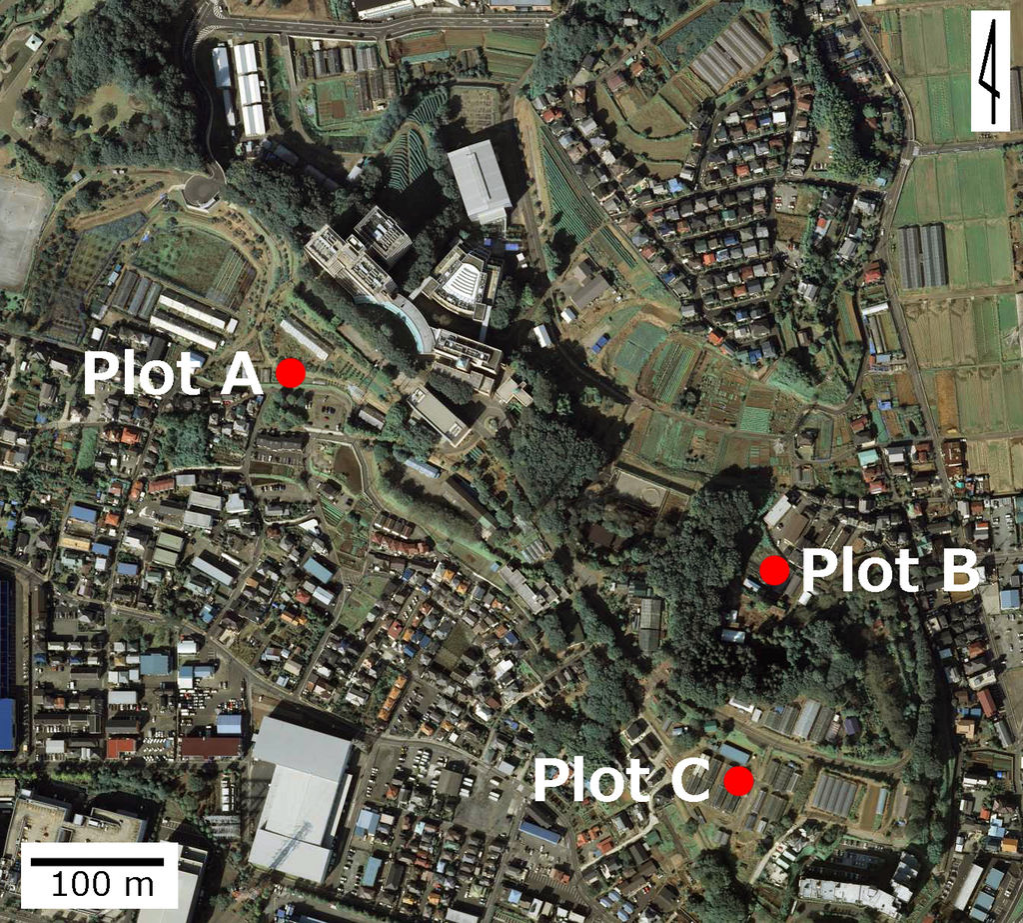
Aerial photograph of the Atsugi Campus of Tokyo University of Agriculture (TUA) and surrounding residential quarters, with locations of the three survey plots (taken in 2007 by the Geospatial Information Authority of Japan).

**Figure 2.**
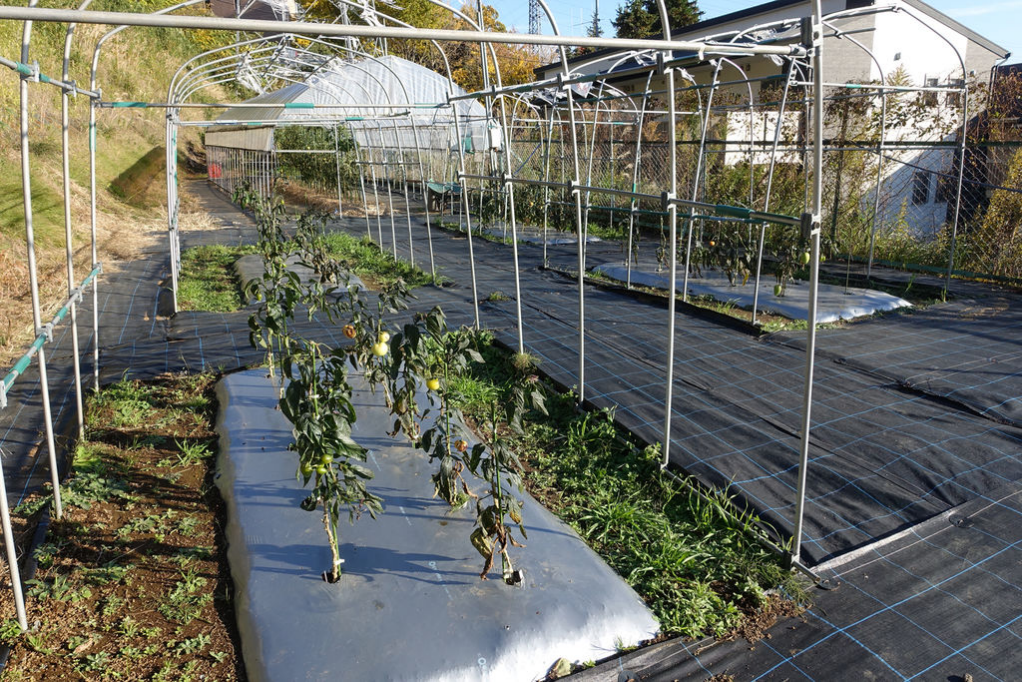
An open field, one of the survey plots in the Atsugi Campus of Tokyo University of Agriculture (TUA), indicated as Plot A in Fig. 1

**Figure 3.**
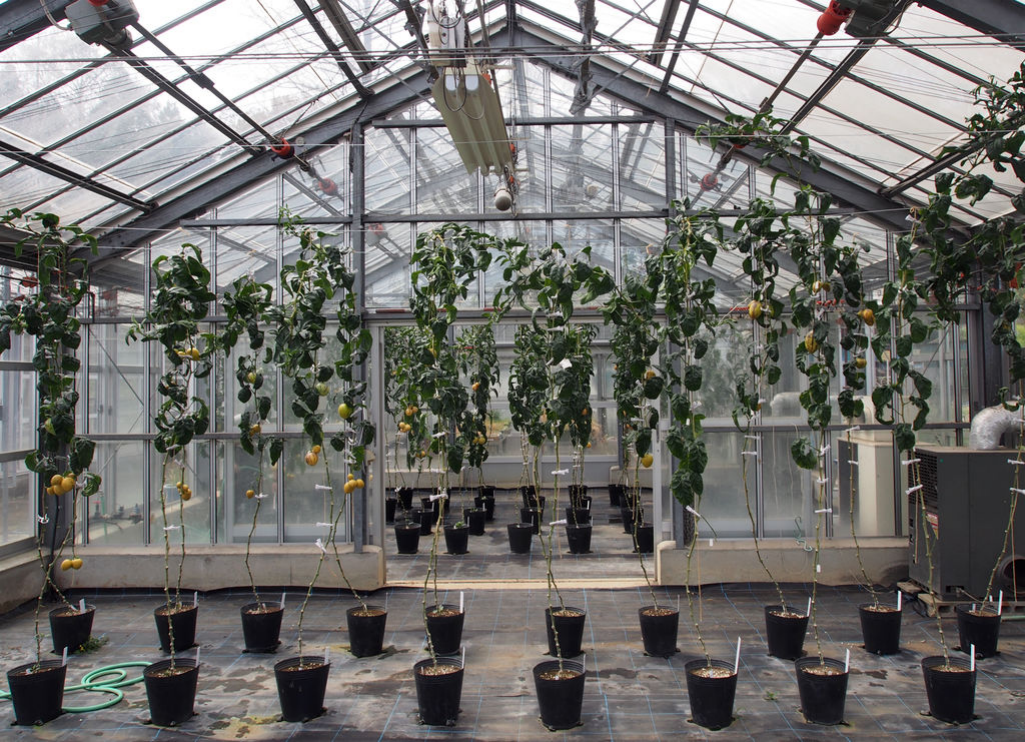
A greenhouse, one of the survey plots in the Atsugi Campus of Tokyo University of Agriculture (TUA), indicated as Plot B in Fig. 1

**Figure 4.**
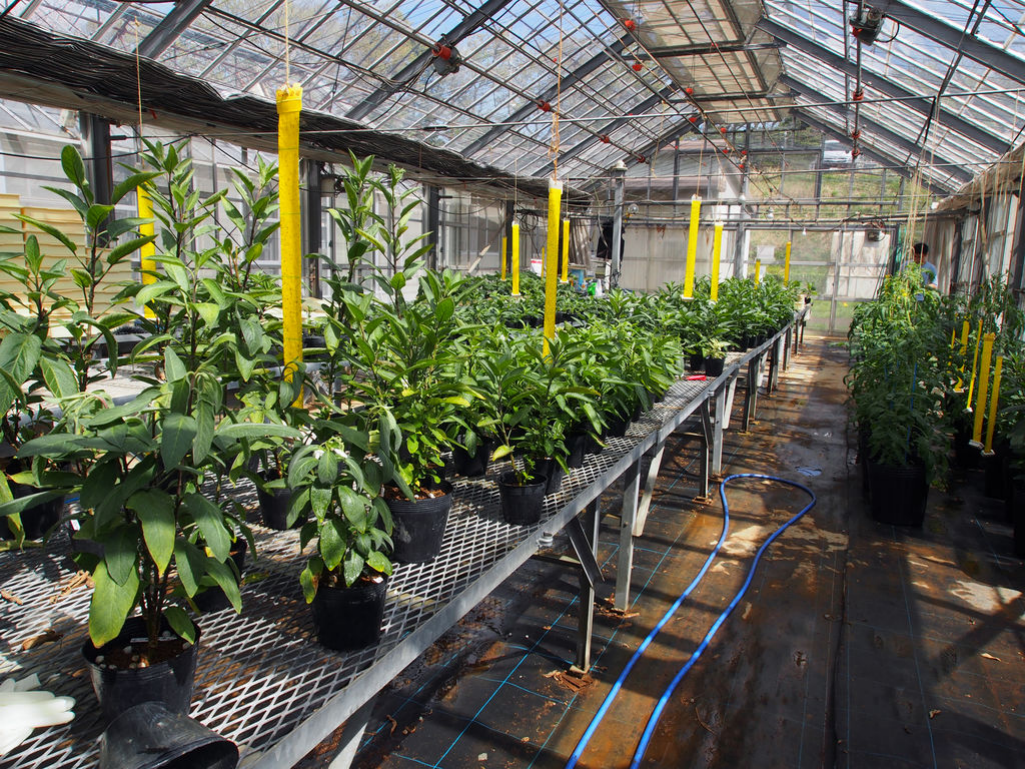
A greenhouse, one of the survey plots in the Atsugi Campus of Tokyo University of Agriculture (TUA), indicated as Plot C in Fig. 1.

This study was conducted at the Atsugi Campus (35.432N 139.346E; at altitudes between 25 and 62 meters above sea level) of Tokyo University of Agriculture (TUA), Atsugi City, Kanagawa Prefecture, Japan, which is surrounded by residential quarters and a woody and grassy park (Fig. 1). The total site area of the campus is approximately 17.3 ha, within which several greenhouses and open fields for experimental use are present. The study site is located in a warm-temperate climate zone and has an annual mean temperature of 15.3 °C and annual mean precipitation of 1,729.9 mm (Japan Meteorological Agency 2017). In the campus, three survey plots were set (Fig. 1); one of these was an open field (approximately 70 m^2^), where 40 pepino plants were cultivated (Plot A) (Fig. 2); another was a greenhouse (approximately 53 m^2^), in which 60 pot pepino plants were grown (Plot B) (Fig. 3); and the other was also a greenhouse (approximately 90 m^2^), in which approximately 400 pot pepino plants were grown (Plot C) (Fig. 4). In these plots, acaricides were applied approximately every two weeks in order to prevent pepino plants from withering due to mites, except for 20 pepino plants in Plot A; when the density of mites became high, appropriate chemicals were sprayed. All the plots were located within a radius of 250 m.

### Sampling methods for insects and mites

All specimens were collected by beating the leaves and branches of pepino plants after observation in field. A total of 34 collections were performed in the three plots from August 30, 2016 to January 21, 2017, for a maximum of 3 h/day in the daytime. The collected insects were killed immediately after capture, using ethyl acetate; aphids, lepidopteran larvae, and mites were fixed in plastic bottles filled with 70–80% ethanol. All specimens were prepared as dry mounted, slide mounded, or ethanol preserved for morphological examination.

### Identification methods for insects and mites

Identification of insect and mite specimens was performed using stereoscopic microscopes (Olympus SZ60 and Olympus SZX16, Tokyo, Japan) and optical microscopes (Olympus BH-2 and Olympus BX41, Tokyo, Japan) by TI and YY according to the following literature: Ehara and Gotoh (2009), Furukawa (2005), Harada and Takizawa (2012), Iwasaki et al. (2000), Kawai (1980), Masumoto and Okajima (2013), Matsumoto (2008), Moritsu (1983), Tanaka and Uesato (2012), Umeya and Okada (2003), Yasunaga et al. (2001), Yasunaga et al. (2015), along with the original descriptions and/or redescriptions of corresponding species if necessary. Collected specimens were regarded as pests in case these were directly damaging insects or mites on pepino plants, were known as pests of pepino in the native range and introduced regions of pepino other than Japan, or were known as pests of major solanaceous crops such as tomato, eggplant, green pepper, and potato, in Japan, with a reference to The Japanese Society of Applied Entomology and Zoology (2006). All examined specimens are preserved in the Insect Collection (IC) at the Laboratory of Entomology, TUA (LETUA).

### Observation of virus-like diseases and virus detection

We surveyed whether pepino plants showed symptoms of virus infection such as mosaic, mottle, necrosis, or chlorosis. The symptomatic leaves were collected and used for virus detection as follows: Total RNA was extracted from the samples using Trizol reagent (Invitrogen Corp., Carlsbad, CA) according to the manufacturer’s instructions. Total RNA was used as a template for first-strand cDNA synthesis by ReverTra Ace -α-**^®^** kit (TOYOBO Co., Ltd., Osaka, Japan) followed by DNA amplification using TaKaRa Ex Taq^TM^ PCR buffer (Takara Bio Inc., Otsu, Japan) with genus-specific or species-specific primers (Table 1). *Alfalfa mosaic virus* (AMV, genus *Alfamovirus*) and *Cucumber mosaic virus* (CMV, genus *Cucumovirus*) had been reported from pepino plants grown in Kanagawa Prefecture, Japan, additionally, *Pepino mosaic virus* (PeMV, genus *Potexvirus*) and two carlaviruses (Potato virus H (PVH) named tentatively and *Potato virus S* (PVS) reclassified) had been detected from symptomatic and asymptomatic pepino plants in abroad, respectively. Even though any potyvirus had not been reported from pepino plants so far, we tried here detecting whether it was occurred or not from our samples. Reverse transcription-polymerase chain reaction (RT-PCR) products were analyzed by electrophoresis in a 2% agarose gel.

## Data resources

In this study, a total of 498 specimens of insects and mites were collected from pepino plants on the three studied plots. Of these specimens, 459 individuals belonging to 11 species were recognized as pests of pepino. They consisted of nine insect species belonging to eight families of five orders and two mite species in two families of one order (Table 2). The remaining specimens (39 individuals) were identified as predatory mites attacking other mites (Acari: Phytoseiidae), parasitic wasps of certain insects (Hymenoptera: Braconidae), and incidental visitors to pepino plants, including grasshoppers (Orthoptera: Tettigoniidae), seed bugs (Hemiptera: Lygaeoidea), and butterflies (Lepidoptera: Papilionidae).

Of these 11 pest species, six were detected from Plot A, three from Plot B, and eight from Plot C (Table 2). The plant bug species *Campylomma livida* Reuter, 1885 was found only in the open field (Plot A). Five species, the cotton whitefly *Bemisia tabaci* (Gennadius, 1889), the mealy bug *Phenacoccus solani* Ferris, 1918, the tomato leaf miner *Liriomyza sativae* Blanchard, 1938, the plusiine noctuid caterpillar *Trichoplusia ni* (Hübner, 1803), and the broad mite *Polyphagotarsonemus latus* (Banks, 1904), were found only in greenhouses (Plots B and C); the former two are well known as glasshouse pests in Japan. No pest species were common in all the three plots. On an empirical basis, through our survey in the plots, two pest species, the two-spotted spider mite *Tetranychus urticae* Koch, 1836 and the cotton aphid *Aphis gossypii* Glover, 1877, were much more abundant on pepino plants than the other pest species recognized, with several hundreds of these two species on each pepino plant.

**Figure 5.**
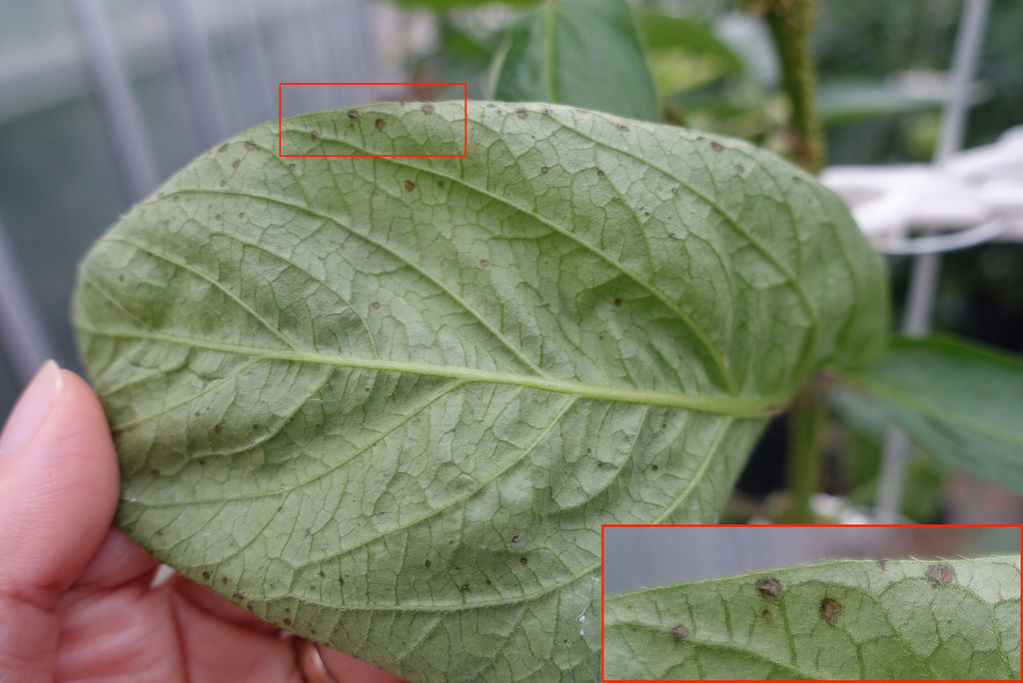
Virus-like symptoms on pepino plants: necrotic spots on pepino leaves in a greenhouse (Fig. 1, Plot C).

**Figure 6.**
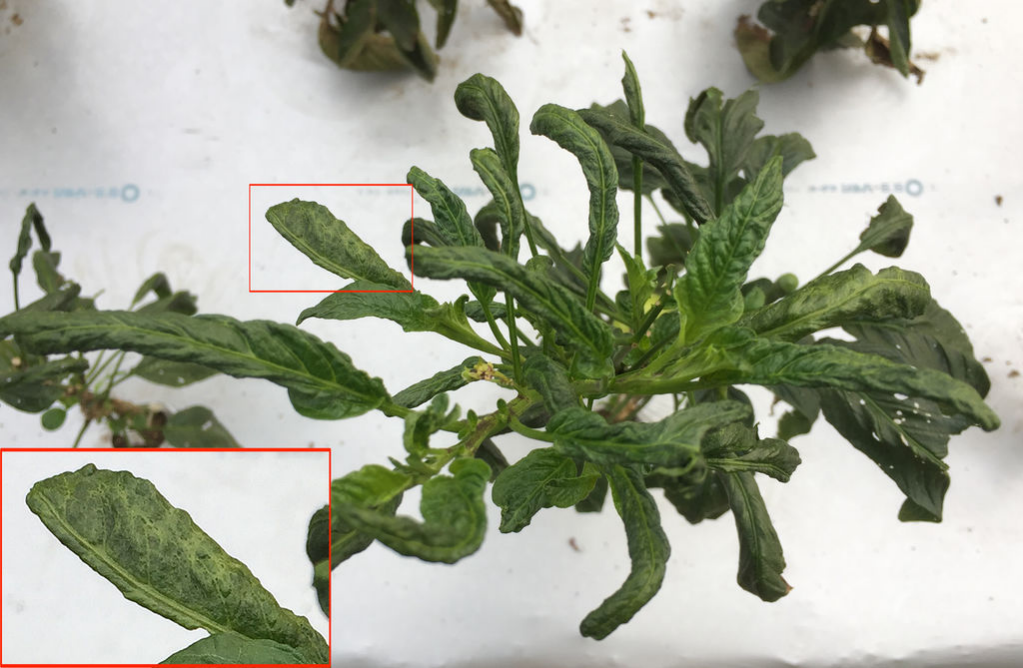
Virus-like symptoms on pepino plants: mottle and deformation on young pepino leaves at an open field (Fig. 1, Plot A).

Regarding virus-like diseases in our research fields, pepino leaves showing necrosis were rarely found in the greenhouse (Fig. 5). However, upper leaves showing mottle symptoms with deformation were remarkably observed only in the acaricide-untreated pepinos in the open field (Plot A) during September to October (Fig. 6). Those symptomatic leaves were tested for the detection of *Alfalfa mosaic virus* (AMV) and *Cucumber mosaic virus* (CMV), as well as the genera *Carlavirus*, *Potexvirus*, and *Potyvirus*. All tested pepino leaves showed no infection of the above-mentioned viruses (data not shown).

## Checklists

### Checklist of insect and mite pests of pepino in Kanagawa, Japan

#### Insecta

Linnaeus, 1758

#### Thysanoptera

Haliday, 1836

#### Thripidae

Stevens, 1829

#### Frankliniella intonsa

(Trybom, 1895)

##### Materials

- **Type status:** Other material. **Occurrence:** recordedBy: Y. Yamada; individualCount: 1; lifeStage: adult; otherCatalogNumbers: 2017-00005; **Taxon:** namePublishedIn: 1895; kingdom: Animalia; phylum: Arthropoda; class: Insecta; order: Thysanoptera; family: Thripidae; genus: Frankliniella; specificEpithet: intonsa; scientificNameAuthorship: Trybom; **Location:** country: Japan; stateProvince: Kanagawa; municipality: Atsugi-shi; locality: Atsugi Campus, Tokyo University of Agriculture, Funako; minimumElevationInMeters: 49; maximumElevationInMeters: 49; decimalLatitude: 35.431707; decimalLongitude: 139.345165; geodeticDatum: WGS84; **Identification:** identifiedBy: Y. Yamada; dateIdentified: 2017; **Event:** samplingProtocol: beating of leaves and branches (including visual searches); eventDate: 2016-09-29; **Record Level:** institutionCode: LETUA; collectionCode: IC
- **Type status:** Other material. **Occurrence:** recordedBy: Y. Yamada; individualCount: 3; lifeStage: adult; otherCatalogNumbers: 2017-00006 | 2017-00007 | 2017-00008; **Taxon:** namePublishedIn: 1895; kingdom: Animalia; phylum: Arthropoda; class: Insecta; order: Thysanoptera; family: Thripidae; genus: Frankliniella; specificEpithet: intonsa; scientificNameAuthorship: Trybom; **Location:** country: Japan; stateProvince: Kanagawa; municipality: Atsugi-shi; locality: Atsugi Campus, Tokyo University of Agriculture, Funako; minimumElevationInMeters: 42; maximumElevationInMeters: 42; decimalLatitude: 35.428874; decimalLongitude: 139.34929; geodeticDatum: WGS84; **Identification:** identifiedBy: Y. Yamada; dateIdentified: 2017; **Event:** samplingProtocol: beating of leaves and branches (including visual searches); eventDate: 2017-01-07; **Record Level:** institutionCode: LETUA; collectionCode: IC
- **Type status:** Other material. **Occurrence:** recordedBy: Y. Yamada; individualCount: 1; lifeStage: adult; otherCatalogNumbers: 2017-00009; **Taxon:** namePublishedIn: 1895; kingdom: Animalia; phylum: Arthropoda; class: Insecta; order: Thysanoptera; family: Thripidae; genus: Frankliniella; specificEpithet: intonsa; scientificNameAuthorship: Trybom; **Location:** country: Japan; stateProvince: Kanagawa; municipality: Atsugi-shi; locality: Atsugi Campus, Tokyo University of Agriculture, Funako; minimumElevationInMeters: 42; maximumElevationInMeters: 42; decimalLatitude: 35.428874; decimalLongitude: 139.34929; geodeticDatum: WGS84; **Identification:** identifiedBy: Y. Yamada; dateIdentified: 2017; **Event:** samplingProtocol: beating of leaves and branches (including visual searches); eventDate: 2017-01-09; **Record Level:** institutionCode: LETUA; collectionCode: IC

#### Hemiptera

Linnaeus, 1758

#### Aleyrodidae

Westwood, 1840

#### Bemisia tabaci

(Gennadius, 1889)

##### Materials

- **Type status:** Other material. **Occurrence:** recordedBy: K.Takahata; individualCount: 1; lifeStage: adult; otherCatalogNumbers: 2017-00010; **Taxon:** namePublishedIn: 1889; kingdom: Animalia; phylum: Arthropoda; class: Insecta; order: Hemiptera; family: Aleyrodidae; genus: Bemisia; specificEpithet: tabaci; scientificNameAuthorship: Gennadius; **Location:** country: Japan; stateProvince: Kanagawa; municipality: Atsugi-shi; locality: Atsugi Campus, Tokyo University of Agriculture, Funako; minimumElevationInMeters: 27; maximumElevationInMeters: 27; decimalLatitude: 35.43043; decimalLongitude: 139.349516; geodeticDatum: WGS84; **Identification:** identifiedBy: Y. Yamada; dateIdentified: 2017; **Event:** samplingProtocol: beating of leaves and branches (including visual searches); eventDate: 2016-12-18; **Record Level:** institutionCode: LETUA; collectionCode: IC
- **Type status:** Other material. **Occurrence:** recordedBy: Y. Yamada; individualCount: 3; lifeStage: adult; otherCatalogNumbers: 2017-00011 | 2017-00012 | 2017-00013; **Taxon:** namePublishedIn: 1889; kingdom: Animalia; phylum: Arthropoda; class: Insecta; order: Hemiptera; family: Aleyrodidae; genus: Bemisia; specificEpithet: tabaci; scientificNameAuthorship: Gennadius; **Location:** country: Japan; stateProvince: Kanagawa; municipality: Atsugi-shi; locality: Atsugi Campus, Tokyo University of Agriculture, Funako; minimumElevationInMeters: 42; maximumElevationInMeters: 42; decimalLatitude: 35.428874; decimalLongitude: 139.34929; geodeticDatum: WGS84; **Identification:** identifiedBy: Y. Yamada; dateIdentified: 2017; **Event:** samplingProtocol: beating of leaves and branches (including visual searches); eventDate: 2016-12-19; **Record Level:** institutionCode: LETUA; collectionCode: IC
- **Type status:** Other material. **Occurrence:** recordedBy: Y. Yamada; individualCount: 2; lifeStage: adult; otherCatalogNumbers: 2017-00014 | 2017-00015; **Taxon:** namePublishedIn: 1889; kingdom: Animalia; phylum: Arthropoda; class: Insecta; order: Hemiptera; family: Aleyrodidae; genus: Bemisia; specificEpithet: tabaci; scientificNameAuthorship: Gennadius; **Location:** country: Japan; stateProvince: Kanagawa; municipality: Atsugi-shi; locality: Atsugi Campus, Tokyo University of Agriculture, Funako; minimumElevationInMeters: 42; maximumElevationInMeters: 42; decimalLatitude: 35.428874; decimalLongitude: 139.34929; geodeticDatum: WGS84; **Identification:** identifiedBy: Y. Yamada; dateIdentified: 2017; **Event:** samplingProtocol: beating of leaves and branches (including visual searches); eventDate: 2017-01-09; **Record Level:** institutionCode: LETUA; collectionCode: IC

#### Aphididae

Latreille, 1802

#### Aphis gossypii

Glover, 1877

##### Materials

- **Type status:** Other material. **Occurrence:** recordedBy: T. Ishikawa; individualCount: 3; lifeStage: adult; otherCatalogNumbers: 2017-00016 | 2017-00017 | 2017-00018; **Taxon:** namePublishedIn: 1877; kingdom: Animalia; phylum: Arthropoda; class: Insecta; order: Hemiptera; family: Aphididae; genus: Aphis; specificEpithet: gossypii; scientificNameAuthorship: Glover; **Location:** country: Japan; stateProvince: Kanagawa; municipality: Atsugi-shi; locality: Atsugi Campus, Tokyo University of Agriculture, Funako; minimumElevationInMeters: 49; maximumElevationInMeters: 49; decimalLatitude: 35.431707; decimalLongitude: 139.345165; geodeticDatum: WGS84; **Identification:** identifiedBy: Y. Yamada; dateIdentified: 2017; **Event:** samplingProtocol: beating of leaves and branches (including visual searches); eventDate: 2016-08-30; **Record Level:** institutionCode: LETUA; collectionCode: IC
- **Type status:** Other material. **Occurrence:** recordedBy: Y. Yamada; individualCount: 3; lifeStage: adult; otherCatalogNumbers: 2017-00019 | 2017-00020 | 2017-00021; **Taxon:** namePublishedIn: 1877; kingdom: Animalia; phylum: Arthropoda; class: Insecta; order: Hemiptera; family: Aphididae; genus: Aphis; specificEpithet: gossypii; scientificNameAuthorship: Glover; **Location:** country: Japan; stateProvince: Kanagawa; municipality: Atsugi-shi; locality: Atsugi Campus, Tokyo University of Agriculture, Funako; minimumElevationInMeters: 49; maximumElevationInMeters: 49; decimalLatitude: 35.431707; decimalLongitude: 139.345165; geodeticDatum: WGS84; **Identification:** identifiedBy: Y. Yamada; dateIdentified: 2017; **Event:** samplingProtocol: beating of leaves and branches (including visual searches); eventDate: 2016-09-28; **Record Level:** institutionCode: LETUA; collectionCode: IC
- **Type status:** Other material. **Occurrence:** recordedBy: Y. Yamada; individualCount: 1; lifeStage: adult; otherCatalogNumbers: 2017-00022; **Taxon:** namePublishedIn: 1877; kingdom: Animalia; phylum: Arthropoda; class: Insecta; order: Hemiptera; family: Aphididae; genus: Aphis; specificEpithet: gossypii; scientificNameAuthorship: Glover; **Location:** country: Japan; stateProvince: Kanagawa; municipality: Atsugi-shi; locality: Atsugi Campus, Tokyo University of Agriculture, Funako; minimumElevationInMeters: 49; maximumElevationInMeters: 49; decimalLatitude: 35.431707; decimalLongitude: 139.345165; geodeticDatum: WGS84; **Identification:** identifiedBy: Y. Yamada; dateIdentified: 2017; **Event:** samplingProtocol: beating of leaves and branches (including visual searches); eventDate: 2016-10-06; **Record Level:** institutionCode: LETUA; collectionCode: IC
- **Type status:** Other material. **Occurrence:** recordedBy: Y. Yamada; individualCount: 1; lifeStage: adult; otherCatalogNumbers: 2017-00023; **Taxon:** namePublishedIn: 1877; kingdom: Animalia; phylum: Arthropoda; class: Insecta; order: Hemiptera; family: Aphididae; genus: Aphis; specificEpithet: gossypii; scientificNameAuthorship: Glover; **Location:** country: Japan; stateProvince: Kanagawa; municipality: Atsugi-shi; locality: Atsugi Campus, Tokyo University of Agriculture, Funako; minimumElevationInMeters: 49; maximumElevationInMeters: 49; decimalLatitude: 35.431707; decimalLongitude: 139.345165; geodeticDatum: WGS84; **Identification:** identifiedBy: Y. Yamada; dateIdentified: 2017; **Event:** samplingProtocol: beating of leaves and branches (including visual searches); eventDate: 2016-11-08; **Record Level:** institutionCode: LETUA; collectionCode: IC
- **Type status:** Other material. **Occurrence:** recordedBy: Y. Yamada; individualCount: 1; lifeStage: adult; otherCatalogNumbers: 2017-00024; **Taxon:** namePublishedIn: 1877; kingdom: Animalia; phylum: Arthropoda; class: Insecta; order: Hemiptera; family: Aphididae; genus: Aphis; specificEpithet: gossypii; scientificNameAuthorship: Glover; **Location:** country: Japan; stateProvince: Kanagawa; municipality: Atsugi-shi; locality: Atsugi Campus, Tokyo University of Agriculture, Funako; minimumElevationInMeters: 49; maximumElevationInMeters: 49; decimalLatitude: 35.431707; decimalLongitude: 139.345165; geodeticDatum: WGS84; **Identification:** identifiedBy: Y. Yamada; dateIdentified: 2017; **Event:** samplingProtocol: beating of leaves and branches (including visual searches); eventDate: 2016-11-10; **Record Level:** institutionCode: LETUA; collectionCode: IC
- **Type status:** Other material. **Occurrence:** recordedBy: Y. Yamada; individualCount: 1; lifeStage: adult; otherCatalogNumbers: 2017-00025; **Taxon:** namePublishedIn: 1877; kingdom: Animalia; phylum: Arthropoda; class: Insecta; order: Hemiptera; family: Aphididae; genus: Aphis; specificEpithet: gossypii; scientificNameAuthorship: Glover; **Location:** country: Japan; stateProvince: Kanagawa; municipality: Atsugi-shi; locality: Atsugi Campus, Tokyo University of Agriculture, Funako; minimumElevationInMeters: 49; maximumElevationInMeters: 49; decimalLatitude: 35.431707; decimalLongitude: 139.345165; geodeticDatum: WGS84; **Identification:** identifiedBy: Y. Yamada; dateIdentified: 2017; **Event:** samplingProtocol: beating of leaves and branches (including visual searches); eventDate: 2016-11-17; **Record Level:** institutionCode: LETUA; collectionCode: IC
- **Type status:** Other material. **Occurrence:** recordedBy: Y. Yamada; individualCount: 4; lifeStage: adult; otherCatalogNumbers: 2017-00026 | 2017-00027 | 2017-00028 | 2017-00029; **Taxon:** namePublishedIn: 1877; kingdom: Animalia; phylum: Arthropoda; class: Insecta; order: Hemiptera; family: Aphididae; genus: Aphis; specificEpithet: gossypii; scientificNameAuthorship: Glover; **Location:** country: Japan; stateProvince: Kanagawa; municipality: Atsugi-shi; locality: Atsugi Campus, Tokyo University of Agriculture, Funako; minimumElevationInMeters: 49; maximumElevationInMeters: 49; decimalLatitude: 35.431707; decimalLongitude: 139.345165; geodeticDatum: WGS84; **Identification:** identifiedBy: Y. Yamada; dateIdentified: 2017; **Event:** samplingProtocol: beating of leaves and branches (including visual searches); eventDate: 2016-11-30; **Record Level:** institutionCode: LETUA; collectionCode: IC
- **Type status:** Other material. **Occurrence:** recordedBy: Y. Yamada; individualCount: 17; lifeStage: adult; otherCatalogNumbers: 2017-00030 | 2017-00031 | 2017-00032 | 2017-00033 | 2017-00034 | 2017-00035 | 2017-00036 | 2017-00037 | 2017-00038 | 2017-00039 | 2017-00040 | 2017-00041 | 2017-00042 | 2017-00043 | 2017-00044 | 2017-00047 | 2017-00048; **Taxon:** namePublishedIn: 1877; kingdom: Animalia; phylum: Arthropoda; class: Insecta; order: Hemiptera; family: Aphididae; genus: Aphis; specificEpithet: gossypii; scientificNameAuthorship: Glover; **Location:** country: Japan; stateProvince: Kanagawa; municipality: Atsugi-shi; locality: Atsugi Campus, Tokyo University of Agriculture, Funako; minimumElevationInMeters: 42; maximumElevationInMeters: 42; decimalLatitude: 35.428874; decimalLongitude: 139.34929; geodeticDatum: WGS84; **Identification:** identifiedBy: Y. Yamada; dateIdentified: 2017; **Event:** samplingProtocol: beating of leaves and branches (including visual searches); eventDate: 2016-12-26; **Record Level:** institutionCode: LETUA; collectionCode: IC
- **Type status:** Other material. **Occurrence:** recordedBy: Y. Yamada; individualCount: 2; lifeStage: nymph; otherCatalogNumbers: 2017-00045 | 2017-00046; **Taxon:** namePublishedIn: 1877; kingdom: Animalia; phylum: Arthropoda; class: Insecta; order: Hemiptera; family: Aphididae; genus: Aphis; specificEpithet: gossypii; scientificNameAuthorship: Glover; **Location:** country: Japan; stateProvince: Kanagawa; municipality: Atsugi-shi; locality: Atsugi Campus, Tokyo University of Agriculture, Funako; minimumElevationInMeters: 42; maximumElevationInMeters: 42; decimalLatitude: 35.428874; decimalLongitude: 139.34929; geodeticDatum: WGS84; **Identification:** identifiedBy: Y. Yamada; dateIdentified: 2017; **Event:** samplingProtocol: beating of leaves and branches (including visual searches); eventDate: 2016-12-26; **Record Level:** institutionCode: LETUA; collectionCode: IC
- **Type status:** Other material. **Occurrence:** recordedBy: Y. Yamada; individualCount: 30; lifeStage: adult; otherCatalogNumbers: 2017-00049 | 2017-00050 | 2017-00051 | 2017-00052 | 2017-00053 | 2017-00054 | 2017-00055 | 2017-00056 | 2017-00057 | 2017-00058 | 2017-00059 | 2017-00060 | 2017-00061 | 2017-00062 | 2017-00063 | 2017-00064 | 2017-00065 | 2017-00066 | 2017-00067 | 2017-00068 | 2017-00069 | 2017-00070 | 2017-00071 | 2017-00072 | 2017-00073 | 2017-00074 | 2017-00075 | 2017-00078 | 2017-00079 | 2017-00080; **Taxon:** namePublishedIn: 1877; kingdom: Animalia; phylum: Arthropoda; class: Insecta; order: Hemiptera; family: Aphididae; genus: Aphis; specificEpithet: gossypii; scientificNameAuthorship: Glover; **Location:** country: Japan; stateProvince: Kanagawa; municipality: Atsugi-shi; locality: Atsugi Campus, Tokyo University of Agriculture, Funako; minimumElevationInMeters: 42; maximumElevationInMeters: 42; decimalLatitude: 35.428874; decimalLongitude: 139.34929; geodeticDatum: WGS84; **Identification:** identifiedBy: Y. Yamada; dateIdentified: 2017; **Event:** samplingProtocol: beating of leaves and branches (including visual searches); eventDate: 2016-12-30; **Record Level:** institutionCode: LETUA; collectionCode: IC
- **Type status:** Other material. **Occurrence:** recordedBy: Y. Yamada; individualCount: 2; lifeStage: nymph; otherCatalogNumbers: 2017-00076 | 2017-00077; **Taxon:** namePublishedIn: 1877; kingdom: Animalia; phylum: Arthropoda; class: Insecta; order: Hemiptera; family: Aphididae; genus: Aphis; specificEpithet: gossypii; scientificNameAuthorship: Glover; **Location:** country: Japan; stateProvince: Kanagawa; municipality: Atsugi-shi; locality: Atsugi Campus, Tokyo University of Agriculture, Funako; minimumElevationInMeters: 42; maximumElevationInMeters: 42; decimalLatitude: 35.428874; decimalLongitude: 139.34929; geodeticDatum: WGS84; **Identification:** identifiedBy: Y. Yamada; dateIdentified: 2017; **Event:** samplingProtocol: beating of leaves and branches (including visual searches); eventDate: 2016-12-30; **Record Level:** institutionCode: LETUA; collectionCode: IC
- **Type status:** Other material. **Occurrence:** recordedBy: Y. Yamada; individualCount: 48; lifeStage: adult; otherCatalogNumbers: 2017-00081 | 2017-00082 | 2017-00083 | 2017-00084 | 2017-00085 | 2017-00086 | 2017-00087 | 2017-00088 | 2017-00089 | 2017-00090 | 2017-00091 | 2017-00092 | 2017-00093 | 2017-00094 | 2017-00095 | 2017-00096 | 2017-00097 | 2017-00098 | 2017-00099 | 2017-00100 | 2017-00101 | 2017-00102 | 2017-00103 | 2017-00104 | 2017-00105 | 2017-00106 | 2017-00107 | 2017-00108 | 2017-00109 | 2017-00110 | 2017-00111 | 2017-00112 | 2017-00113 | 2017-00114 | 2017-00115 | 2017-00116 | 2017-00117 | 2017-00118 | 2017-00119 | 2017-00120 | 2017-00121 | 2017-00122 | 2017-00131 | 2017-00132 | 2017-00133 | 2017-00134 | 2017-00135 | 2017-00136; **Taxon:** namePublishedIn: 1877; kingdom: Animalia; phylum: Arthropoda; class: Insecta; order: Hemiptera; family: Aphididae; genus: Aphis; specificEpithet: gossypii; scientificNameAuthorship: Glover; **Location:** country: Japan; stateProvince: Kanagawa; municipality: Atsugi-shi; locality: Atsugi Campus, Tokyo University of Agriculture, Funako; minimumElevationInMeters: 42; maximumElevationInMeters: 42; decimalLatitude: 35.428874; decimalLongitude: 139.34929; geodeticDatum: WGS84; **Identification:** identifiedBy: Y. Yamada; dateIdentified: 2017; **Event:** samplingProtocol: beating of leaves and branches (including visual searches); eventDate: 2017-01-03; **Record Level:** institutionCode: LETUA; collectionCode: IC
- **Type status:** Other material. **Occurrence:** recordedBy: Y. Yamada; individualCount: 8; lifeStage: nymph; otherCatalogNumbers: 2017-00123 | 2017-00124 | 2017-00125 | 2017-00126 | 2017-00127 | 2017-00128 | 2017-00129 | 2017-00130; **Taxon:** namePublishedIn: 1877; kingdom: Animalia; phylum: Arthropoda; class: Insecta; order: Hemiptera; family: Aphididae; genus: Aphis; specificEpithet: gossypii; scientificNameAuthorship: Glover; **Location:** country: Japan; stateProvince: Kanagawa; municipality: Atsugi-shi; locality: Atsugi Campus, Tokyo University of Agriculture, Funako; minimumElevationInMeters: 42; maximumElevationInMeters: 42; decimalLatitude: 35.428874; decimalLongitude: 139.34929; geodeticDatum: WGS84; **Identification:** identifiedBy: Y. Yamada; dateIdentified: 2017; **Event:** samplingProtocol: beating of leaves and branches (including visual searches); eventDate: 2017-01-03; **Record Level:** institutionCode: LETUA; collectionCode: IC
- **Type status:** Other material. **Occurrence:** recordedBy: Y. Yamada; individualCount: 36; lifeStage: adult; otherCatalogNumbers: 2017-00137 | 2017-00138 | 2017-00139 | 2017-00140 | 2017-00141 | 2017-00142 | 2017-00143 | 2017-00144 | 2017-00145 | 2017-00146 | 2017-00147 | 2017-00148 | 2017-00149 | 2017-00150 | 2017-00151 | 2017-00152 | 2017-00153 | 2017-00154 | 2017-00155 | 2017-00156 | 2017-00157 | 2017-00158 | 2017-00159 | 2017-00160 | 2017-00161 | 2017-00162 | 2017-00163 | 2017-00164 | 2017-00165 | 2017-00166 | 2017-00167 | 2017-00168 | 2017-00169 | 2017-00170 | 2017-00171 | 2017-00172; **Taxon:** namePublishedIn: 1877; kingdom: Animalia; phylum: Arthropoda; class: Insecta; order: Hemiptera; family: Aphididae; genus: Aphis; specificEpithet: gossypii; scientificNameAuthorship: Glover; **Location:** country: Japan; stateProvince: Kanagawa; municipality: Atsugi-shi; locality: Atsugi Campus, Tokyo University of Agriculture, Funako; minimumElevationInMeters: 42; maximumElevationInMeters: 42; decimalLatitude: 35.428874; decimalLongitude: 139.34929; geodeticDatum: WGS84; **Identification:** identifiedBy: Y. Yamada; dateIdentified: 2017; **Event:** samplingProtocol: beating of leaves and branches (including visual searches); eventDate: 2017-01-05; **Record Level:** institutionCode: LETUA; collectionCode: IC
- **Type status:** Other material. **Occurrence:** recordedBy: Y. Yamada; individualCount: 25; lifeStage: adult; otherCatalogNumbers: 2017-00173 | 2017-00174 | 2017-00175 | 2017-00176 | 2017-00177 | 2017-00178 | 2017-00179 | 2017-00180 | 2017-00181 | 2017-00182 | 2017-00183 | 2017-00184 | 2017-00185 | 2017-00186 | 2017-00187 | 2017-00188 | 2017-00189 | 2017-00190 | 2017-00191 | 2017-00192 | 2017-00193 | 2017-00194 | 2017-00195 | 2017-00196 | 2017-00197; **Taxon:** namePublishedIn: 1877; kingdom: Animalia; phylum: Arthropoda; class: Insecta; order: Hemiptera; family: Aphididae; genus: Aphis; specificEpithet: gossypii; scientificNameAuthorship: Glover; **Location:** country: Japan; stateProvince: Kanagawa; municipality: Atsugi-shi; locality: Atsugi Campus, Tokyo University of Agriculture, Funako; minimumElevationInMeters: 42; maximumElevationInMeters: 42; decimalLatitude: 35.428874; decimalLongitude: 139.34929; geodeticDatum: WGS84; **Identification:** identifiedBy: Y. Yamada; dateIdentified: 2017; **Event:** samplingProtocol: beating of leaves and branches (including visual searches); eventDate: 2017-01-07; **Record Level:** institutionCode: LETUA; collectionCode: IC
- **Type status:** Other material. **Occurrence:** recordedBy: Y. Yamada; individualCount: 4; lifeStage: adult; otherCatalogNumbers: 2017-00198 | 2017-00199 | 2017-00200 | 2017-00201; **Taxon:** namePublishedIn: 1877; kingdom: Animalia; phylum: Arthropoda; class: Insecta; order: Hemiptera; family: Aphididae; genus: Aphis; specificEpithet: gossypii; scientificNameAuthorship: Glover; **Location:** country: Japan; stateProvince: Kanagawa; municipality: Atsugi-shi; locality: Atsugi Campus, Tokyo University of Agriculture, Funako; minimumElevationInMeters: 42; maximumElevationInMeters: 42; decimalLatitude: 35.428874; decimalLongitude: 139.34929; geodeticDatum: WGS84; **Identification:** identifiedBy: Y. Yamada; dateIdentified: 2017; **Event:** samplingProtocol: beating of leaves and branches (including visual searches); eventDate: 2017-01-09; **Record Level:** institutionCode: LETUA; collectionCode: IC
- **Type status:** Other material. **Occurrence:** recordedBy: Y. Yamada; individualCount: 43; lifeStage: adult; otherCatalogNumbers: 2017-00202 | 2017-00203 | 2017-00204 | 2017-00205 | 2017-00206 | 2017-00207 | 2017-00208 | 2017-00209 | 2017-00210 | 2017-00211 | 2017-00212 | 2017-00213 | 2017-00214 | 2017-00215 | 2017-00216 | 2017-00217 | 2017-00218 | 2017-00219 | 2017-00220 | 2017-00221 | 2017-00222 | 2017-00223 | 2017-00224 | 2017-00225 | 2017-00226 | 2017-00227 | 2017-00228 | 2017-00229 | 2017-00230 | 2017-00231 | 2017-00232 | 2017-00233 | 2017-00234 | 2017-00235 | 2017-00236 | 2017-00237 | 2017-00238 | 2017-00239 | 2017-00240 | 2017-00241 | 2017-00242 | 2017-00243 | 2017-00244; **Taxon:** namePublishedIn: 1877; kingdom: Animalia; phylum: Arthropoda; class: Insecta; order: Hemiptera; family: Aphididae; genus: Aphis; specificEpithet: gossypii; scientificNameAuthorship: Glover; **Location:** country: Japan; stateProvince: Kanagawa; municipality: Atsugi-shi; locality: Atsugi Campus, Tokyo University of Agriculture, Funako; minimumElevationInMeters: 42; maximumElevationInMeters: 42; decimalLatitude: 35.428874; decimalLongitude: 139.34929; geodeticDatum: WGS84; **Identification:** identifiedBy: Y. Yamada; dateIdentified: 2017; **Event:** samplingProtocol: beating of leaves and branches (including visual searches); eventDate: 2017-01-12; **Record Level:** institutionCode: LETUA; collectionCode: IC
- **Type status:** Other material. **Occurrence:** recordedBy: Y. Yamada; individualCount: 22; lifeStage: adult; otherCatalogNumbers: 2017-00245 | 2017-00246 | 2017-00247 | 2017-00248 | 2017-00249 | 2017-00250 | 2017-00251 | 2017-00252 | 2017-00253 | 2017-00254 | 2017-00255 | 2017-00256 | 2017-00257 | 2017-00258 | 2017-00259 | 2017-00260 | 2017-00261 | 2017-00262 | 2017-00263 | 2017-00264 | 2017-00265 | 2017-00266; **Taxon:** namePublishedIn: 1877; kingdom: Animalia; phylum: Arthropoda; class: Insecta; order: Hemiptera; family: Aphididae; genus: Aphis; specificEpithet: gossypii; scientificNameAuthorship: Glover; **Location:** country: Japan; stateProvince: Kanagawa; municipality: Atsugi-shi; locality: Atsugi Campus, Tokyo University of Agriculture, Funako; minimumElevationInMeters: 42; maximumElevationInMeters: 42; decimalLatitude: 35.428874; decimalLongitude: 139.34929; geodeticDatum: WGS84; **Identification:** identifiedBy: Y. Yamada; dateIdentified: 2017; **Event:** samplingProtocol: beating of leaves and branches (including visual searches); eventDate: 2017-01-16; **Record Level:** institutionCode: LETUA; collectionCode: IC
- **Type status:** Other material. **Occurrence:** recordedBy: Y. Yamada; individualCount: 3; lifeStage: adult; otherCatalogNumbers: 2017-00267 | 2017-00268 | 2017-00269; **Taxon:** namePublishedIn: 1877; kingdom: Animalia; phylum: Arthropoda; class: Insecta; order: Hemiptera; family: Aphididae; genus: Aphis; specificEpithet: gossypii; scientificNameAuthorship: Glover; **Location:** country: Japan; stateProvince: Kanagawa; municipality: Atsugi-shi; locality: Atsugi Campus, Tokyo University of Agriculture, Funako; minimumElevationInMeters: 42; maximumElevationInMeters: 42; decimalLatitude: 35.428874; decimalLongitude: 139.34929; geodeticDatum: WGS84; **Identification:** identifiedBy: Y. Yamada; dateIdentified: 2017; **Event:** samplingProtocol: beating of leaves and branches (including visual searches); eventDate: 2017-01-21; **Record Level:** institutionCode: LETUA; collectionCode: IC

#### Pseudococcidae

Heymons, 1915

#### Phenacoccus solani

Ferris, 1918

##### Materials

- **Type status:** Other material. **Occurrence:** recordedBy: Y. Yamada; individualCount: 1; lifeStage: nymph; otherCatalogNumbers: 2017-00270; **Taxon:** namePublishedIn: 1918; kingdom: Animalia; phylum: Arthropoda; class: Insecta; order: Hemiptera; family: Pseudococcidae; genus: Phenacoccus; specificEpithet: solani; scientificNameAuthorship: Ferris; **Location:** country: Japan; stateProvince: Kanagawa; municipality: Atsugi-shi; locality: Atsugi Campus, Tokyo University of Agriculture, Funako; minimumElevationInMeters: 42; maximumElevationInMeters: 42; decimalLatitude: 35.428874; decimalLongitude: 139.34929; geodeticDatum: WGS84; **Identification:** identifiedBy: Y. Yamada; dateIdentified: 2017; **Event:** samplingProtocol: beating of leaves and branches (including visual searches); eventDate: 2016-12-30; **Record Level:** institutionCode: LETUA; collectionCode: IC
- **Type status:** Other material. **Occurrence:** recordedBy: Y. Yamada; individualCount: 2; lifeStage: adult; otherCatalogNumbers: 2017-00271 | 2017-00272; **Taxon:** namePublishedIn: 1918; kingdom: Animalia; phylum: Arthropoda; class: Insecta; order: Hemiptera; family: Pseudococcidae; genus: Phenacoccus; specificEpithet: solani; scientificNameAuthorship: Ferris; **Location:** country: Japan; stateProvince: Kanagawa; municipality: Atsugi-shi; locality: Atsugi Campus, Tokyo University of Agriculture, Funako; minimumElevationInMeters: 42; maximumElevationInMeters: 42; decimalLatitude: 35.428874; decimalLongitude: 139.34929; geodeticDatum: WGS84; **Identification:** identifiedBy: Y. Yamada; dateIdentified: 2017; **Event:** samplingProtocol: beating of leaves and branches (including visual searches); eventDate: 2017-01-15; **Record Level:** institutionCode: LETUA; collectionCode: IC

#### Miridae

Hahn, 1833

#### Campylomma livida

Reuter, 1885

##### Materials

- **Type status:** Other material. **Occurrence:** recordedBy: Y. Yamada; individualCount: 1; lifeStage: nymph; otherCatalogNumbers: 2017-00273; **Taxon:** namePublishedIn: 1885; kingdom: Animalia; phylum: Arthropoda; class: Insecta; order: Hemiptera; family: Miridae; genus: Campylomma; specificEpithet: livida; scientificNameAuthorship: Reuter; **Location:** country: Japan; stateProvince: Kanagawa; municipality: Atsugi-shi; locality: Atsugi Campus, Tokyo University of Agriculture, Funako; minimumElevationInMeters: 49; maximumElevationInMeters: 49; decimalLatitude: 35.431707; decimalLongitude: 139.345165; geodeticDatum: WGS84; **Identification:** identifiedBy: Y. Yamada; dateIdentified: 2017; **Event:** samplingProtocol: beating of leaves and branches (including visual searches); eventDate: 2016-11-10; **Record Level:** institutionCode: LETUA; collectionCode: IC
- **Type status:** Other material. **Occurrence:** recordedBy: Y. Yamada; individualCount: 1; lifeStage: adult; otherCatalogNumbers: 2017-00274; **Taxon:** namePublishedIn: 1885; kingdom: Animalia; phylum: Arthropoda; class: Insecta; order: Hemiptera; family: Miridae; genus: Campylomma; specificEpithet: livida; scientificNameAuthorship: Reuter; **Location:** country: Japan; stateProvince: Kanagawa; municipality: Atsugi-shi; locality: Atsugi Campus, Tokyo University of Agriculture, Funako; minimumElevationInMeters: 49; maximumElevationInMeters: 49; decimalLatitude: 35.431707; decimalLongitude: 139.345165; geodeticDatum: WGS84; **Identification:** identifiedBy: Y. Yamada; dateIdentified: 2017; **Event:** samplingProtocol: beating of leaves and branches (including visual searches); eventDate: 2016-11-10; **Record Level:** institutionCode: LETUA; collectionCode: IC
- **Type status:** Other material. **Occurrence:** recordedBy: Y. Yamada; individualCount: 1; lifeStage: adult; otherCatalogNumbers: 2017-00275; **Taxon:** namePublishedIn: 1885; kingdom: Animalia; phylum: Arthropoda; class: Insecta; order: Hemiptera; family: Miridae; genus: Campylomma; specificEpithet: livida; scientificNameAuthorship: Reuter; **Location:** country: Japan; stateProvince: Kanagawa; municipality: Atsugi-shi; locality: Atsugi Campus, Tokyo University of Agriculture, Funako; minimumElevationInMeters: 49; maximumElevationInMeters: 49; decimalLatitude: 35.431707; decimalLongitude: 139.345165; geodeticDatum: WGS84; **Identification:** identifiedBy: Y. Yamada; dateIdentified: 2017; **Event:** samplingProtocol: beating of leaves and branches (including visual searches); eventDate: 2016-11-15; **Record Level:** institutionCode: LETUA; collectionCode: IC
- **Type status:** Other material. **Occurrence:** recordedBy: Y. Yamada; individualCount: 1; lifeStage: adult; otherCatalogNumbers: 2017-00276; **Taxon:** namePublishedIn: 1885; kingdom: Animalia; phylum: Arthropoda; class: Insecta; order: Hemiptera; family: Miridae; genus: Campylomma; specificEpithet: livida; scientificNameAuthorship: Reuter; **Location:** country: Japan; stateProvince: Kanagawa; municipality: Atsugi-shi; locality: Atsugi Campus, Tokyo University of Agriculture, Funako; minimumElevationInMeters: 49; maximumElevationInMeters: 49; decimalLatitude: 35.431707; decimalLongitude: 139.345165; geodeticDatum: WGS84; **Identification:** identifiedBy: Y. Yamada; dateIdentified: 2017; **Event:** samplingProtocol: beating of leaves and branches (including visual searches); eventDate: 2016-11-17; **Record Level:** institutionCode: LETUA; collectionCode: IC
- **Type status:** Other material. **Occurrence:** recordedBy: Y. Yamada; individualCount: 1; lifeStage: nymph; otherCatalogNumbers: 2017-00277; **Taxon:** namePublishedIn: 1885; kingdom: Animalia; phylum: Arthropoda; class: Insecta; order: Hemiptera; family: Miridae; genus: Campylomma; specificEpithet: livida; scientificNameAuthorship: Reuter; **Location:** country: Japan; stateProvince: Kanagawa; municipality: Atsugi-shi; locality: Atsugi Campus, Tokyo University of Agriculture, Funako; minimumElevationInMeters: 49; maximumElevationInMeters: 49; decimalLatitude: 35.431707; decimalLongitude: 139.345165; geodeticDatum: WGS84; **Identification:** identifiedBy: Y. Yamada; dateIdentified: 2017; **Event:** samplingProtocol: beating of leaves and branches (including visual searches); eventDate: 2016-11-17; **Record Level:** institutionCode: LETUA; collectionCode: IC
- **Type status:** Other material. **Occurrence:** recordedBy: Y. Yamada; individualCount: 1; lifeStage: nymph; otherCatalogNumbers: 2017-00278; **Taxon:** namePublishedIn: 1885; kingdom: Animalia; phylum: Arthropoda; class: Insecta; order: Hemiptera; family: Miridae; genus: Campylomma; specificEpithet: livida; scientificNameAuthorship: Reuter; **Location:** country: Japan; stateProvince: Kanagawa; municipality: Atsugi-shi; locality: Atsugi Campus, Tokyo University of Agriculture, Funako; minimumElevationInMeters: 49; maximumElevationInMeters: 49; decimalLatitude: 35.431707; decimalLongitude: 139.345165; geodeticDatum: WGS84; **Identification:** identifiedBy: Y. Yamada; dateIdentified: 2017; **Event:** samplingProtocol: beating of leaves and branches (including visual searches); eventDate: 2016-11-23; **Record Level:** institutionCode: LETUA; collectionCode: IC
- **Type status:** Other material. **Occurrence:** recordedBy: Y. Yamada; individualCount: 1; lifeStage: adult; otherCatalogNumbers: 2017-00279; **Taxon:** namePublishedIn: 1885; kingdom: Animalia; phylum: Arthropoda; class: Insecta; order: Hemiptera; family: Miridae; genus: Campylomma; specificEpithet: livida; scientificNameAuthorship: Reuter; **Location:** country: Japan; stateProvince: Kanagawa; municipality: Atsugi-shi; locality: Atsugi Campus, Tokyo University of Agriculture, Funako; minimumElevationInMeters: 49; maximumElevationInMeters: 49; decimalLatitude: 35.431707; decimalLongitude: 139.345165; geodeticDatum: WGS84; **Identification:** identifiedBy: Y. Yamada; dateIdentified: 2017; **Event:** samplingProtocol: beating of leaves and branches (including visual searches); eventDate: 2016-11-23; **Record Level:** institutionCode: LETUA; collectionCode: IC
- **Type status:** Other material. **Occurrence:** recordedBy: Y. Yamada; individualCount: 1; lifeStage: adult; otherCatalogNumbers: 2017-00280; **Taxon:** namePublishedIn: 1885; kingdom: Animalia; phylum: Arthropoda; class: Insecta; order: Hemiptera; family: Miridae; genus: Campylomma; specificEpithet: livida; scientificNameAuthorship: Reuter; **Location:** country: Japan; stateProvince: Kanagawa; municipality: Atsugi-shi; locality: Atsugi Campus, Tokyo University of Agriculture, Funako; minimumElevationInMeters: 49; maximumElevationInMeters: 49; decimalLatitude: 35.431707; decimalLongitude: 139.345165; geodeticDatum: WGS84; **Identification:** identifiedBy: Y. Yamada; dateIdentified: 2017; **Event:** samplingProtocol: beating of leaves and branches (including visual searches); eventDate: 2016-11-25; **Record Level:** institutionCode: LETUA; collectionCode: IC
- **Type status:** Other material. **Occurrence:** recordedBy: Y. Yamada; individualCount: 1; lifeStage: adult; otherCatalogNumbers: 2017-00281; **Taxon:** namePublishedIn: 1885; kingdom: Animalia; phylum: Arthropoda; class: Insecta; order: Hemiptera; family: Miridae; genus: Campylomma; specificEpithet: livida; scientificNameAuthorship: Reuter; **Location:** country: Japan; stateProvince: Kanagawa; municipality: Atsugi-shi; locality: Atsugi Campus, Tokyo University of Agriculture, Funako; minimumElevationInMeters: 49; maximumElevationInMeters: 49; decimalLatitude: 35.431707; decimalLongitude: 139.345165; geodeticDatum: WGS84; **Identification:** identifiedBy: Y. Yamada; dateIdentified: 2017; **Event:** samplingProtocol: beating of leaves and branches (including visual searches); eventDate: 2016-11-30; **Record Level:** institutionCode: LETUA; collectionCode: IC
- **Type status:** Other material. **Occurrence:** recordedBy: Y. Yamada; individualCount: 2; lifeStage: nymph; otherCatalogNumbers: 2017-00282 | 2017-00283; **Taxon:** namePublishedIn: 1885; kingdom: Animalia; phylum: Arthropoda; class: Insecta; order: Hemiptera; family: Miridae; genus: Campylomma; specificEpithet: livida; scientificNameAuthorship: Reuter; **Location:** country: Japan; stateProvince: Kanagawa; municipality: Atsugi-shi; locality: Atsugi Campus, Tokyo University of Agriculture, Funako; minimumElevationInMeters: 49; maximumElevationInMeters: 49; decimalLatitude: 35.431707; decimalLongitude: 139.345165; geodeticDatum: WGS84; **Identification:** identifiedBy: Y. Yamada; dateIdentified: 2017; **Event:** samplingProtocol: beating of leaves and branches (including visual searches); eventDate: 2016-11-30; **Record Level:** institutionCode: LETUA; collectionCode: IC

#### Coleoptera

Linnaeus, 1758

#### Chrysomelidae

Latreille, 1802

#### Epitrix hirtipennis

(Melsheimer, 1847)

##### Materials

- **Type status:** Other material. **Occurrence:** recordedBy: T. Ishikawa; individualCount: 12; lifeStage: adult; otherCatalogNumbers: 2017-00284 | 2017-00285 | 2017-00286 | 2017-00287 | 2017-00288 | 2017-00289 | 2017-00290 | 2017-00291 | 2017-00292 | 2017-00293 | 2017-00294 | 2017-00295; **Taxon:** namePublishedIn: 1847; kingdom: Animalia; phylum: Arthropoda; class: Insecta; order: Coleoptera; family: Chrysomelidae; genus: Epitrix; specificEpithet: hirtipennis; scientificNameAuthorship: Melsheimer; **Location:** country: Japan; stateProvince: Kanagawa; municipality: Atsugi-shi; locality: Atsugi Campus, Tokyo University of Agriculture, Funako; minimumElevationInMeters: 49; maximumElevationInMeters: 49; decimalLatitude: 35.431707; decimalLongitude: 139.345165; geodeticDatum: WGS84; **Identification:** identifiedBy: Y. Yamada; dateIdentified: 2017; **Event:** samplingProtocol: beating of leaves and branches (including visual searches); eventDate: 2016-08-30; **Record Level:** institutionCode: LETUA; collectionCode: IC
- **Type status:** Other material. **Occurrence:** recordedBy: Y. Yamada; individualCount: 4; lifeStage: adult; otherCatalogNumbers: 2017-00296 | 2017-00297 | 2017-00298 | 2017-00299; **Taxon:** namePublishedIn: 1847; kingdom: Animalia; phylum: Arthropoda; class: Insecta; order: Coleoptera; family: Chrysomelidae; genus: Epitrix; specificEpithet: hirtipennis; scientificNameAuthorship: Melsheimer; **Location:** country: Japan; stateProvince: Kanagawa; municipality: Atsugi-shi; locality: Atsugi Campus, Tokyo University of Agriculture, Funako; minimumElevationInMeters: 49; maximumElevationInMeters: 49; decimalLatitude: 35.431707; decimalLongitude: 139.345165; geodeticDatum: WGS84; **Identification:** identifiedBy: Y. Yamada; dateIdentified: 2017; **Event:** samplingProtocol: beating of leaves and branches (including visual searches); eventDate: 2016-09-28; **Record Level:** institutionCode: LETUA; collectionCode: IC
- **Type status:** Other material. **Occurrence:** recordedBy: Y. Yamada; individualCount: 5; lifeStage: adult; otherCatalogNumbers: 2017-00300 | 2017-00301 | 2017-00302 | 2017-00303 | 2017-00304; **Taxon:** namePublishedIn: 1847; kingdom: Animalia; phylum: Arthropoda; class: Insecta; order: Coleoptera; family: Chrysomelidae; genus: Epitrix; specificEpithet: hirtipennis; scientificNameAuthorship: Melsheimer; **Location:** country: Japan; stateProvince: Kanagawa; municipality: Atsugi-shi; locality: Atsugi Campus, Tokyo University of Agriculture, Funako; minimumElevationInMeters: 49; maximumElevationInMeters: 49; decimalLatitude: 35.431707; decimalLongitude: 139.345165; geodeticDatum: WGS84; **Identification:** identifiedBy: Y. Yamada; dateIdentified: 2017; **Event:** samplingProtocol: beating of leaves and branches (including visual searches); eventDate: 2016-09-29; **Record Level:** institutionCode: LETUA; collectionCode: IC
- **Type status:** Other material. **Occurrence:** recordedBy: Y. Yamada; individualCount: 8; lifeStage: adult; otherCatalogNumbers: 2017-00305 | 2017-00306 | 2017-00307 | 2017-00308 | 2017-00309 | 2017-00310 | 2017-00311 | 2017-00312; **Taxon:** namePublishedIn: 1847; kingdom: Animalia; phylum: Arthropoda; class: Insecta; order: Coleoptera; family: Chrysomelidae; genus: Epitrix; specificEpithet: hirtipennis; scientificNameAuthorship: Melsheimer; **Location:** country: Japan; stateProvince: Kanagawa; municipality: Atsugi-shi; locality: Atsugi Campus, Tokyo University of Agriculture, Funako; minimumElevationInMeters: 49; maximumElevationInMeters: 49; decimalLatitude: 35.431707; decimalLongitude: 139.345165; geodeticDatum: WGS84; **Identification:** identifiedBy: Y. Yamada; dateIdentified: 2017; **Event:** samplingProtocol: beating of leaves and branches (including visual searches); eventDate: 2016-09-30; **Record Level:** institutionCode: LETUA; collectionCode: IC
- **Type status:** Other material. **Occurrence:** recordedBy: Y. Yamada; individualCount: 7; lifeStage: adult; otherCatalogNumbers: 2017-00313 | 2017-00314 | 2017-00315 | 2017-00316 | 2017-00317 | 2017-00318 | 2017-00319; **Taxon:** namePublishedIn: 1847; kingdom: Animalia; phylum: Arthropoda; class: Insecta; order: Coleoptera; family: Chrysomelidae; genus: Epitrix; specificEpithet: hirtipennis; scientificNameAuthorship: Melsheimer; **Location:** country: Japan; stateProvince: Kanagawa; municipality: Atsugi-shi; locality: Atsugi Campus, Tokyo University of Agriculture, Funako; minimumElevationInMeters: 49; maximumElevationInMeters: 49; decimalLatitude: 35.431707; decimalLongitude: 139.345165; geodeticDatum: WGS84; **Identification:** identifiedBy: Y. Yamada; dateIdentified: 2017; **Event:** samplingProtocol: beating of leaves and branches (including visual searches); eventDate: 2016-10-01; **Record Level:** institutionCode: LETUA; collectionCode: IC
- **Type status:** Other material. **Occurrence:** recordedBy: Y. Yamada; individualCount: 4; lifeStage: adult; otherCatalogNumbers: 2017-00320 | 2017-00321 | 2017-00322 | 2017-00323; **Taxon:** namePublishedIn: 1847; kingdom: Animalia; phylum: Arthropoda; class: Insecta; order: Coleoptera; family: Chrysomelidae; genus: Epitrix; specificEpithet: hirtipennis; scientificNameAuthorship: Melsheimer; **Location:** country: Japan; stateProvince: Kanagawa; municipality: Atsugi-shi; locality: Atsugi Campus, Tokyo University of Agriculture, Funako; minimumElevationInMeters: 49; maximumElevationInMeters: 49; decimalLatitude: 35.431707; decimalLongitude: 139.345165; geodeticDatum: WGS84; **Identification:** identifiedBy: Y. Yamada; dateIdentified: 2017; **Event:** samplingProtocol: beating of leaves and branches (including visual searches); eventDate: 2016-10-04; **Record Level:** institutionCode: LETUA; collectionCode: IC
- **Type status:** Other material. **Occurrence:** recordedBy: Y. Yamada; individualCount: 4; lifeStage: adult; otherCatalogNumbers: 2017-00324 | 2017-00325 | 2017-00326 | 2017-00327; **Taxon:** namePublishedIn: 1847; kingdom: Animalia; phylum: Arthropoda; class: Insecta; order: Coleoptera; family: Chrysomelidae; genus: Epitrix; specificEpithet: hirtipennis; scientificNameAuthorship: Melsheimer; **Location:** country: Japan; stateProvince: Kanagawa; municipality: Atsugi-shi; locality: Atsugi Campus, Tokyo University of Agriculture, Funako; minimumElevationInMeters: 49; maximumElevationInMeters: 49; decimalLatitude: 35.431707; decimalLongitude: 139.345165; geodeticDatum: WGS84; **Identification:** identifiedBy: Y. Yamada; dateIdentified: 2017; **Event:** samplingProtocol: beating of leaves and branches (including visual searches); eventDate: 2016-10-14; **Record Level:** institutionCode: LETUA; collectionCode: IC
- **Type status:** Other material. **Occurrence:** recordedBy: Y. Yamada; individualCount: 4; lifeStage: adult; otherCatalogNumbers: 2017-00328 | 2017-00329 | 2017-00330 | 2017-00331; **Taxon:** namePublishedIn: 1847; kingdom: Animalia; phylum: Arthropoda; class: Insecta; order: Coleoptera; family: Chrysomelidae; genus: Epitrix; specificEpithet: hirtipennis; scientificNameAuthorship: Melsheimer; **Location:** country: Japan; stateProvince: Kanagawa; municipality: Atsugi-shi; locality: Atsugi Campus, Tokyo University of Agriculture, Funako; minimumElevationInMeters: 49; maximumElevationInMeters: 49; decimalLatitude: 35.431707; decimalLongitude: 139.345165; geodeticDatum: WGS84; **Identification:** identifiedBy: Y. Yamada; dateIdentified: 2017; **Event:** samplingProtocol: beating of leaves and branches (including visual searches); eventDate: 2016-10-15; **Record Level:** institutionCode: LETUA; collectionCode: IC
- **Type status:** Other material. **Occurrence:** recordedBy: Y. Yamada; individualCount: 8; lifeStage: adult; otherCatalogNumbers: 2017-00332 | 2017-00333 | 2017-00334 | 2017-00335 | 2017-00336 | 2017-00337 | 2017-00338 | 2017-00339; **Taxon:** namePublishedIn: 1847; kingdom: Animalia; phylum: Arthropoda; class: Insecta; order: Coleoptera; family: Chrysomelidae; genus: Epitrix; specificEpithet: hirtipennis; scientificNameAuthorship: Melsheimer; **Location:** country: Japan; stateProvince: Kanagawa; municipality: Atsugi-shi; locality: Atsugi Campus, Tokyo University of Agriculture, Funako; minimumElevationInMeters: 49; maximumElevationInMeters: 49; decimalLatitude: 35.431707; decimalLongitude: 139.345165; geodeticDatum: WGS84; **Identification:** identifiedBy: Y. Yamada; dateIdentified: 2017; **Event:** samplingProtocol: beating of leaves and branches (including visual searches); eventDate: 2016-10-18; **Record Level:** institutionCode: LETUA; collectionCode: IC
- **Type status:** Other material. **Occurrence:** recordedBy: Y. Yamada; individualCount: 1; lifeStage: adult; otherCatalogNumbers: 2017-00340; **Taxon:** namePublishedIn: 1847; kingdom: Animalia; phylum: Arthropoda; class: Insecta; order: Coleoptera; family: Chrysomelidae; genus: Epitrix; specificEpithet: hirtipennis; scientificNameAuthorship: Melsheimer; **Location:** country: Japan; stateProvince: Kanagawa; municipality: Atsugi-shi; locality: Atsugi Campus, Tokyo University of Agriculture, Funako; minimumElevationInMeters: 49; maximumElevationInMeters: 49; decimalLatitude: 35.431707; decimalLongitude: 139.345165; geodeticDatum: WGS84; **Identification:** identifiedBy: Y. Yamada; dateIdentified: 2017; **Event:** samplingProtocol: beating of leaves and branches (including visual searches); eventDate: 2016-10-20; **Record Level:** institutionCode: LETUA; collectionCode: IC
- **Type status:** Other material. **Occurrence:** recordedBy: Y. Yamada; individualCount: 1; lifeStage: adult; otherCatalogNumbers: 2017-00341; **Taxon:** namePublishedIn: 1847; kingdom: Animalia; phylum: Arthropoda; class: Insecta; order: Coleoptera; family: Chrysomelidae; genus: Epitrix; specificEpithet: hirtipennis; scientificNameAuthorship: Melsheimer; **Location:** country: Japan; stateProvince: Kanagawa; municipality: Atsugi-shi; locality: Atsugi Campus, Tokyo University of Agriculture, Funako; minimumElevationInMeters: 49; maximumElevationInMeters: 49; decimalLatitude: 35.431707; decimalLongitude: 139.345165; geodeticDatum: WGS84; **Identification:** identifiedBy: Y. Yamada; dateIdentified: 2017; **Event:** samplingProtocol: beating of leaves and branches (including visual searches); eventDate: 2016-10-24; **Record Level:** institutionCode: LETUA; collectionCode: IC
- **Type status:** Other material. **Occurrence:** recordedBy: Y. Yamada; individualCount: 4; lifeStage: adult; otherCatalogNumbers: 2017-00342 | 2017-00343 | 2017-00344 | 2017-00345; **Taxon:** namePublishedIn: 1847; kingdom: Animalia; phylum: Arthropoda; class: Insecta; order: Coleoptera; family: Chrysomelidae; genus: Epitrix; specificEpithet: hirtipennis; scientificNameAuthorship: Melsheimer; **Location:** country: Japan; stateProvince: Kanagawa; municipality: Atsugi-shi; locality: Atsugi Campus, Tokyo University of Agriculture, Funako; minimumElevationInMeters: 49; maximumElevationInMeters: 49; decimalLatitude: 35.431707; decimalLongitude: 139.345165; geodeticDatum: WGS84; **Identification:** identifiedBy: Y. Yamada; dateIdentified: 2017; **Event:** samplingProtocol: beating of leaves and branches (including visual searches); eventDate: 2016-11-03; **Record Level:** institutionCode: LETUA; collectionCode: IC
- **Type status:** Other material. **Occurrence:** recordedBy: Y. Yamada; individualCount: 2; lifeStage: adult; otherCatalogNumbers: 2017-00346 | 2017-00347; **Taxon:** namePublishedIn: 1847; kingdom: Animalia; phylum: Arthropoda; class: Insecta; order: Coleoptera; family: Chrysomelidae; genus: Epitrix; specificEpithet: hirtipennis; scientificNameAuthorship: Melsheimer; **Location:** country: Japan; stateProvince: Kanagawa; municipality: Atsugi-shi; locality: Atsugi Campus, Tokyo University of Agriculture, Funako; minimumElevationInMeters: 49; maximumElevationInMeters: 49; decimalLatitude: 35.431707; decimalLongitude: 139.345165; geodeticDatum: WGS84; **Identification:** identifiedBy: Y. Yamada; dateIdentified: 2017; **Event:** samplingProtocol: beating of leaves and branches (including visual searches); eventDate: 2016-11-08; **Record Level:** institutionCode: LETUA; collectionCode: IC
- **Type status:** Other material. **Occurrence:** recordedBy: Y. Yamada; individualCount: 1; lifeStage: adult; otherCatalogNumbers: 2017-00348; **Taxon:** namePublishedIn: 1847; kingdom: Animalia; phylum: Arthropoda; class: Insecta; order: Coleoptera; family: Chrysomelidae; genus: Epitrix; specificEpithet: hirtipennis; scientificNameAuthorship: Melsheimer; **Location:** country: Japan; stateProvince: Kanagawa; municipality: Atsugi-shi; locality: Atsugi Campus, Tokyo University of Agriculture, Funako; minimumElevationInMeters: 49; maximumElevationInMeters: 49; decimalLatitude: 35.431707; decimalLongitude: 139.345165; geodeticDatum: WGS84; **Identification:** identifiedBy: Y. Yamada; dateIdentified: 2017; **Event:** samplingProtocol: beating of leaves and branches (including visual searches); eventDate: 2016-11-10; **Record Level:** institutionCode: LETUA; collectionCode: IC
- **Type status:** Other material. **Occurrence:** recordedBy: Y. Yamada; individualCount: 1; lifeStage: adult; otherCatalogNumbers: 2017-00349; **Taxon:** namePublishedIn: 1847; kingdom: Animalia; phylum: Arthropoda; class: Insecta; order: Coleoptera; family: Chrysomelidae; genus: Epitrix; specificEpithet: hirtipennis; scientificNameAuthorship: Melsheimer; **Location:** country: Japan; stateProvince: Kanagawa; municipality: Atsugi-shi; locality: Atsugi Campus, Tokyo University of Agriculture, Funako; minimumElevationInMeters: 49; maximumElevationInMeters: 49; decimalLatitude: 35.431707; decimalLongitude: 139.345165; geodeticDatum: WGS84; **Identification:** identifiedBy: Y. Yamada; dateIdentified: 2017; **Event:** samplingProtocol: beating of leaves and branches (including visual searches); eventDate: 2016-11-15; **Record Level:** institutionCode: LETUA; collectionCode: IC
- **Type status:** Other material. **Occurrence:** recordedBy: Y. Yamada; individualCount: 1; lifeStage: adult; otherCatalogNumbers: 2017-00350; **Taxon:** namePublishedIn: 1847; kingdom: Animalia; phylum: Arthropoda; class: Insecta; order: Coleoptera; family: Chrysomelidae; genus: Epitrix; specificEpithet: hirtipennis; scientificNameAuthorship: Melsheimer; **Location:** country: Japan; stateProvince: Kanagawa; municipality: Atsugi-shi; locality: Atsugi Campus, Tokyo University of Agriculture, Funako; minimumElevationInMeters: 49; maximumElevationInMeters: 49; decimalLatitude: 35.431707; decimalLongitude: 139.345165; geodeticDatum: WGS84; **Identification:** identifiedBy: Y. Yamada; dateIdentified: 2017; **Event:** samplingProtocol: beating of leaves and branches (including visual searches); eventDate: 2016-11-17; **Record Level:** institutionCode: LETUA; collectionCode: IC
- **Type status:** Other material. **Occurrence:** recordedBy: Y. Yamada; individualCount: 2; lifeStage: adult; otherCatalogNumbers: 2017-00351 | 2017-00352; **Taxon:** namePublishedIn: 1847; kingdom: Animalia; phylum: Arthropoda; class: Insecta; order: Coleoptera; family: Chrysomelidae; genus: Epitrix; specificEpithet: hirtipennis; scientificNameAuthorship: Melsheimer; **Location:** country: Japan; stateProvince: Kanagawa; municipality: Atsugi-shi; locality: Atsugi Campus, Tokyo University of Agriculture, Funako; minimumElevationInMeters: 49; maximumElevationInMeters: 49; decimalLatitude: 35.431707; decimalLongitude: 139.345165; geodeticDatum: WGS84; **Identification:** identifiedBy: Y. Yamada; dateIdentified: 2017; **Event:** samplingProtocol: beating of leaves and branches (including visual searches); eventDate: 2016-11-25; **Record Level:** institutionCode: LETUA; collectionCode: IC

##### Notes

Known as a recent alien species to Japan (Harada and Takizawa 2012)

#### Lepidoptera

Linnaeus, 1758

#### Noctuidae

Latreille, 1809

#### Spodoptera litura

(Fabricius, 1775)

##### Materials

- **Type status:** Other material. **Occurrence:** recordedBy: Y. Yamada; individualCount: 1; lifeStage: larva; otherCatalogNumbers: 2017-00353; **Taxon:** namePublishedIn: 1775; kingdom: Animalia; phylum: Arthropoda; class: Insecta; order: Lepidoptera; family: Noctuidae; genus: Spodoptera; specificEpithet: litura; scientificNameAuthorship: Fabricius; **Location:** country: Japan; stateProvince: Kanagawa; municipality: Atsugi-shi; locality: Atsugi Campus, Tokyo University of Agriculture, Funako; minimumElevationInMeters: 49; maximumElevationInMeters: 49; decimalLatitude: 35.431707; decimalLongitude: 139.345165; geodeticDatum: WGS84; **Identification:** identifiedBy: T. Ishikawa; dateIdentified: 2017; **Event:** samplingProtocol: beating of leaves and branches (including visual searches); eventDate: 2016-10-24; **Record Level:** institutionCode: LETUA; collectionCode: IC
- **Type status:** Other material. **Occurrence:** recordedBy: Y. Yamada; individualCount: 1; lifeStage: larva; otherCatalogNumbers: 2017-00354; **Taxon:** namePublishedIn: 1775; kingdom: Animalia; phylum: Arthropoda; class: Insecta; order: Lepidoptera; family: Noctuidae; genus: Spodoptera; specificEpithet: litura; scientificNameAuthorship: Fabricius; **Location:** country: Japan; stateProvince: Kanagawa; municipality: Atsugi-shi; locality: Atsugi Campus, Tokyo University of Agriculture, Funako; minimumElevationInMeters: 27; maximumElevationInMeters: 27; decimalLatitude: 35.43043; decimalLongitude: 139.349516; geodeticDatum: WGS84; **Identification:** identifiedBy: T. Ishikawa; dateIdentified: 2017; **Event:** samplingProtocol: beating of leaves and branches (including visual searches); eventDate: 2016-11-18; **Record Level:** institutionCode: LETUA; collectionCode: IC

#### Trichoplusia ni

(Hübner, 1803)

##### Materials

- **Type status:** Other material. **Occurrence:** recordedBy: K. Niwa; individualCount: 1; lifeStage: larva; otherCatalogNumbers: 2017-00355; **Taxon:** namePublishedIn: 1803; kingdom: Animalia; phylum: Arthropoda; class: Insecta; order: Lepidoptera; family: Noctuidae; genus: Trichoplusia; specificEpithet: ni; scientificNameAuthorship: Hübner; **Location:** country: Japan; stateProvince: Kanagawa; municipality: Atsugi-shi; locality: Atsugi Campus, Tokyo University of Agriculture, Funako; minimumElevationInMeters: 27; maximumElevationInMeters: 27; decimalLatitude: 35.43043; decimalLongitude: 139.349516; geodeticDatum: WGS84; **Identification:** identifiedBy: T. Ishikawa; dateIdentified: 2017; **Event:** samplingProtocol: beating of leaves and branches (including visual searches); eventDate: 2016-11-08; **Record Level:** institutionCode: LETUA; collectionCode: IC
- **Type status:** Other material. **Occurrence:** recordedBy: K. Niwa; individualCount: 8; lifeStage: larva; otherCatalogNumbers: 2017-00356 | 2017-00357 | 2017-00358 | 2017-00359 | 2017-00360 | 2017-00361 | 2017-00362 | 2017-00363; **Taxon:** namePublishedIn: 1803; kingdom: Animalia; phylum: Arthropoda; class: Insecta; order: Lepidoptera; family: Noctuidae; genus: Trichoplusia; specificEpithet: ni; scientificNameAuthorship: Hübner; **Location:** country: Japan; stateProvince: Kanagawa; municipality: Atsugi-shi; locality: Atsugi Campus, Tokyo University of Agriculture, Funako; minimumElevationInMeters: 27; maximumElevationInMeters: 27; decimalLatitude: 35.43043; decimalLongitude: 139.349516; geodeticDatum: WGS84; **Identification:** identifiedBy: T. Ishikawa; dateIdentified: 2017; **Event:** samplingProtocol: beating of leaves and branches (including visual searches); eventDate: 2016-11-18; **Record Level:** institutionCode: LETUA; collectionCode: IC

#### Diptera

Linnaeus, 1758

#### Agromyzidae

Fallén, 1823

#### Liriomyza sativae

Blanchard, 1938

##### Materials

- **Type status:** Other material. **Occurrence:** recordedBy: O-k. Kim; individualCount: 4; lifeStage: adult; otherCatalogNumbers: 2017-00364 | 2017-00365 | 2017-00366 | 2017-00367; **Taxon:** namePublishedIn: 1938; kingdom: Animalia; phylum: Arthropoda; class: Insecta; order: Diptera; family: Agromyzidae; genus: Liriomyza; specificEpithet: sativae; scientificNameAuthorship: Blanchard; **Location:** country: Japan; stateProvince: Kanagawa; municipality: Atsugi-shi; locality: Atsugi Campus, Tokyo University of Agriculture, Funako; minimumElevationInMeters: 42; maximumElevationInMeters: 42; decimalLatitude: 35.428874; decimalLongitude: 139.34929; geodeticDatum: WGS84; **Identification:** identifiedBy: Y. Yamada; dateIdentified: 2017; **Event:** samplingProtocol: beating of leaves and branches (including visual searches); eventDate: 2016-11-17; **Record Level:** institutionCode: LETUA; collectionCode: IC
- **Type status:** Other material. **Occurrence:** recordedBy: Y. Yamada; individualCount: 5; lifeStage: adult; otherCatalogNumbers: 2017-00368 | 2017-00369 | 2017-00370 | 2017-00371 | 2017-00372; **Taxon:** namePublishedIn: 1938; kingdom: Animalia; phylum: Arthropoda; class: Insecta; order: Diptera; family: Agromyzidae; genus: Liriomyza; specificEpithet: sativae; scientificNameAuthorship: Blanchard; **Location:** country: Japan; stateProvince: Kanagawa; municipality: Atsugi-shi; locality: Atsugi Campus, Tokyo University of Agriculture, Funako; minimumElevationInMeters: 42; maximumElevationInMeters: 42; decimalLatitude: 35.428874; decimalLongitude: 139.34929; geodeticDatum: WGS84; **Identification:** identifiedBy: Y. Yamada; dateIdentified: 2017; **Event:** samplingProtocol: beating of leaves and branches (including visual searches); eventDate: 2016-12-15; **Record Level:** institutionCode: LETUA; collectionCode: IC
- **Type status:** Other material. **Occurrence:** recordedBy: Y. Yamada; individualCount: 1; lifeStage: adult; otherCatalogNumbers: 2017-00373; **Taxon:** namePublishedIn: 1938; kingdom: Animalia; phylum: Arthropoda; class: Insecta; order: Diptera; family: Agromyzidae; genus: Liriomyza; specificEpithet: sativae; scientificNameAuthorship: Blanchard; **Location:** country: Japan; stateProvince: Kanagawa; municipality: Atsugi-shi; locality: Atsugi Campus, Tokyo University of Agriculture, Funako; minimumElevationInMeters: 42; maximumElevationInMeters: 42; decimalLatitude: 35.428874; decimalLongitude: 139.34929; geodeticDatum: WGS84; **Identification:** identifiedBy: Y. Yamada; dateIdentified: 2017; **Event:** samplingProtocol: beating of leaves and branches (including visual searches); eventDate: 2016-12-19; **Record Level:** institutionCode: LETUA; collectionCode: IC
- **Type status:** Other material. **Occurrence:** recordedBy: Y. Yamada; individualCount: 13; lifeStage: adult; otherCatalogNumbers: 2017-00374 | 2017-00375 | 2017-00376 | 2017-00377 | 2017-00378 | 2017-00379 | 2017-00380 | 2017-00381 | 2017-00382 | 2017-00383 | 2017-00384 | 2017-00385 | 2017-00386; **Taxon:** namePublishedIn: 1938; kingdom: Animalia; phylum: Arthropoda; class: Insecta; order: Diptera; family: Agromyzidae; genus: Liriomyza; specificEpithet: sativae; scientificNameAuthorship: Blanchard; **Location:** country: Japan; stateProvince: Kanagawa; municipality: Atsugi-shi; locality: Atsugi Campus, Tokyo University of Agriculture, Funako; minimumElevationInMeters: 42; maximumElevationInMeters: 42; decimalLatitude: 35.428874; decimalLongitude: 139.34929; geodeticDatum: WGS84; **Identification:** identifiedBy: Y. Yamada; dateIdentified: 2017; **Event:** samplingProtocol: beating of leaves and branches (including visual searches); eventDate: 2017-01-07; **Record Level:** institutionCode: LETUA; collectionCode: IC

#### Arachnida

Lamarck, 1801

#### Acari

Leach, 1817

#### Tarsonemidae

Kramer, 1877

#### Polyphagotarsonemus latus

(Banks, 1904)

##### Materials

- **Type status:** Other material. **Occurrence:** recordedBy: Y. Yamada; individualCount: 1; lifeStage: adult; otherCatalogNumbers: 2017-00387; **Taxon:** namePublishedIn: 1904; kingdom: Animalia; phylum: Arthropoda; class: Arachnida; order: Acari; family: Tarsonemidae; genus: Polyphagotarsonemus; specificEpithet: latus; scientificNameAuthorship: Banks; **Location:** country: Japan; stateProvince: Kanagawa; municipality: Atsugi-shi; locality: Atsugi Campus, Tokyo University of Agriculture, Funako; minimumElevationInMeters: 42; maximumElevationInMeters: 42; decimalLatitude: 35.428874; decimalLongitude: 139.34929; geodeticDatum: WGS84; **Identification:** identifiedBy: Y. Yamada; dateIdentified: 2017; **Event:** samplingProtocol: beating of leaves and branches (including visual searches); eventDate: 2017-01-07; **Record Level:** institutionCode: LETUA; collectionCode: IC

#### Tetranychidae

Donnadieu, 1875

#### Tetranychus urticae

Koch, 1836

##### Materials

- **Type status:** Other material. **Occurrence:** recordedBy: T. Ishikawa; individualCount: 8; lifeStage: adult; otherCatalogNumbers: 2017-00388 | 2017-00389 | 2017-00390 | 2017-00391 | 2017-00392 | 2017-00393 | 2017-00394 | 2017-00395; **Taxon:** namePublishedIn: 1836; kingdom: Animalia; phylum: Arthropoda; class: Arachnida; order: Acari; family: Tetranychidae; genus: Tetranychus; specificEpithet: urticae; scientificNameAuthorship: Koch; **Location:** country: Japan; stateProvince: Kanagawa; municipality: Atsugi-shi; locality: Atsugi Campus, Tokyo University of Agriculture, Funako; minimumElevationInMeters: 49; maximumElevationInMeters: 49; decimalLatitude: 35.431707; decimalLongitude: 139.345165; geodeticDatum: WGS84; **Identification:** identifiedBy: Y. Yamada; dateIdentified: 2017; **Event:** samplingProtocol: beating of leaves and branches (including visual searches); eventDate: 2016-08-30; **Record Level:** institutionCode: LETUA; collectionCode: IC
- **Type status:** Other material. **Occurrence:** recordedBy: Y. Yamada; individualCount: 13; lifeStage: adult; otherCatalogNumbers: 2017-00396 | 2017-00397 | 2017-00398 | 2017-00399 | 2017-00400 | 2017-00401 | 2017-00402 | 2017-00403 | 2017-00404 | 2017-00405 | 2017-00406 | 2017-00407 | 2017-00408; **Taxon:** namePublishedIn: 1836; kingdom: Animalia; phylum: Arthropoda; class: Arachnida; order: Acari; family: Tetranychidae; genus: Tetranychus; specificEpithet: urticae; scientificNameAuthorship: Koch; **Location:** country: Japan; stateProvince: Kanagawa; municipality: Atsugi-shi; locality: Atsugi Campus, Tokyo University of Agriculture, Funako; minimumElevationInMeters: 42; maximumElevationInMeters: 42; decimalLatitude: 35.428874; decimalLongitude: 139.34929; geodeticDatum: WGS84; **Identification:** identifiedBy: Y. Yamada; dateIdentified: 2017; **Event:** samplingProtocol: beating of leaves and branches (including visual searches); eventDate: 2017-01-07; **Record Level:** institutionCode: LETUA; collectionCode: IC
- **Type status:** Other material. **Occurrence:** recordedBy: Y. Yamada; individualCount: 18; lifeStage: adult; otherCatalogNumbers: 2017-00409 | 2017-00410 | 2017-00411 | 2017-00412 | 2017-00413 | 2017-00414 | 2017-00415 | 2017-00416 | 2017-00417 | 2017-00418 | 2017-00419 | 2017-00420 | 2017-00421 | 2017-00422 | 2017-00423 | 2017-00424 | 2017-00425 | 2017-00426; **Taxon:** namePublishedIn: 1836; kingdom: Animalia; phylum: Arthropoda; class: Arachnida; order: Acari; family: Tetranychidae; genus: Tetranychus; specificEpithet: urticae; scientificNameAuthorship: Koch; **Location:** country: Japan; stateProvince: Kanagawa; municipality: Atsugi-shi; locality: Atsugi Campus, Tokyo University of Agriculture, Funako; minimumElevationInMeters: 42; maximumElevationInMeters: 42; decimalLatitude: 35.428874; decimalLongitude: 139.34929; geodeticDatum: WGS84; **Identification:** identifiedBy: Y. Yamada; dateIdentified: 2017; **Event:** samplingProtocol: beating of leaves and branches (including visual searches); eventDate: 2017-01-09; **Record Level:** institutionCode: LETUA; collectionCode: IC
- **Type status:** Other material. **Occurrence:** recordedBy: Y. Yamada; individualCount: 2; lifeStage: larva; otherCatalogNumbers: 2017-00427 | 2017-00428; **Taxon:** namePublishedIn: 1836; kingdom: Animalia; phylum: Arthropoda; class: Arachnida; order: Acari; family: Tetranychidae; genus: Tetranychus; specificEpithet: urticae; scientificNameAuthorship: Koch; **Location:** country: Japan; stateProvince: Kanagawa; municipality: Atsugi-shi; locality: Atsugi Campus, Tokyo University of Agriculture, Funako; minimumElevationInMeters: 42; maximumElevationInMeters: 42; decimalLatitude: 35.428874; decimalLongitude: 139.34929; geodeticDatum: WGS84; **Identification:** identifiedBy: Y. Yamada; dateIdentified: 2017; **Event:** samplingProtocol: beating of leaves and branches (including visual searches); eventDate: 2017-01-09; **Record Level:** institutionCode: LETUA; collectionCode: IC
- **Type status:** Other material. **Occurrence:** recordedBy: Y. Yamada; individualCount: 15; lifeStage: adult; otherCatalogNumbers: 2017-00429 | 2017-00430 | 2017-00431 | 2017-00432 | 2017-00433 | 2017-00434 | 2017-00435 | 2017-00436 | 2017-00437 | 2017-00438 | 2017-00439 | 2017-00440 | 2017-00441 | 2017-00442 | 2017-00443; **Taxon:** namePublishedIn: 1836; kingdom: Animalia; phylum: Arthropoda; class: Arachnida; order: Acari; family: Tetranychidae; genus: Tetranychus; specificEpithet: urticae; scientificNameAuthorship: Koch; **Location:** country: Japan; stateProvince: Kanagawa; municipality: Atsugi-shi; locality: Atsugi Campus, Tokyo University of Agriculture, Funako; minimumElevationInMeters: 42; maximumElevationInMeters: 42; decimalLatitude: 35.428874; decimalLongitude: 139.34929; geodeticDatum: WGS84; **Identification:** identifiedBy: Y. Yamada; dateIdentified: 2017; **Event:** samplingProtocol: beating of leaves and branches (including visual searches); eventDate: 2017-01-12; **Record Level:** institutionCode: LETUA; collectionCode: IC
- **Type status:** Other material. **Occurrence:** recordedBy: Y. Yamada; individualCount: 8; lifeStage: adult; otherCatalogNumbers: 2017-00444 | 2017-00445 | 2017-00446 | 2017-00447 | 2017-00448 | 2017-00449 | 2017-00450 | 2017-00451; **Taxon:** namePublishedIn: 1836; kingdom: Animalia; phylum: Arthropoda; class: Arachnida; order: Acari; family: Tetranychidae; genus: Tetranychus; specificEpithet: urticae; scientificNameAuthorship: Koch; **Location:** country: Japan; stateProvince: Kanagawa; municipality: Atsugi-shi; locality: Atsugi Campus, Tokyo University of Agriculture, Funako; minimumElevationInMeters: 42; maximumElevationInMeters: 42; decimalLatitude: 35.428874; decimalLongitude: 139.34929; geodeticDatum: WGS84; **Identification:** identifiedBy: Y. Yamada; dateIdentified: 2017; **Event:** samplingProtocol: beating of leaves and branches (including visual searches); eventDate: 2017-01-16; **Record Level:** institutionCode: LETUA; collectionCode: IC
- **Type status:** Other material. **Occurrence:** recordedBy: Y. Yamada; individualCount: 12; lifeStage: adult; otherCatalogNumbers: 2017-00452 | 2017-00453 | 2017-00454 | 2017-00455 | 2017-00456 | 2017-00457 | 2017-00458 | 2017-00459 | 2017-00460 | 2017-00461 | 2017-00462 | 2017-00463; **Taxon:** namePublishedIn: 1836; kingdom: Animalia; phylum: Arthropoda; class: Arachnida; order: Acari; family: Tetranychidae; genus: Tetranychus; specificEpithet: urticae; scientificNameAuthorship: Koch; **Location:** country: Japan; stateProvince: Kanagawa; municipality: Atsugi-shi; locality: Atsugi Campus, Tokyo University of Agriculture, Funako; minimumElevationInMeters: 42; maximumElevationInMeters: 42; decimalLatitude: 35.428874; decimalLongitude: 139.34929; geodeticDatum: WGS84; **Identification:** identifiedBy: Y. Yamada; dateIdentified: 2017; **Event:** samplingProtocol: beating of leaves and branches (including visual searches); eventDate: 2017-01-21; **Record Level:** institutionCode: LETUA; collectionCode: IC

## Discussion

Prior to the present study, nine groups of insects and mites have been known as pests of pepino plants in Japan (Table 3), including those that are inadequately identified, such as *Helicoverpa* spp. at the genus level, aphids at the family level (Aphididae), and mites at the order level (Acari). Among these, three groups were identified at the species level, comprising the greenhouse whitefly *Trialeurodes vaporariorum* (Westwood, 1856), the potato tuberworm *Phthorimaea operculella* (Zeller, 1873), and the two-spotted spider mite *Tetranychus urticae* Koch, 1836. In the present study, our survey in the campus revealed the presence of 11 species of insect and mite pests on pepino as mentioned above. Only *T. urticae* is common in the previous records as well as the results of the present study; in addition, *T. vaporariorum* and *P. operculella* were not found in our survey. Currently, 13 species of insects and mites are recognized as pests of pepino plants in Japan; all of these are also the pests of major solanaceous crops such as tomato, eggplant, green pepper, and potato (The Japanese Society of Applied Entomology and Zoology 2006). It is likely that *T. vaporariorum* and *P. operculella* may be detected on pepino from the campus, because the two species are widely distributed in Kanagawa Prefecture (Matsumoto 2004, Nakajima and Yamamoto 2004) where our study was carried out.

Currently, 25 species of insects and mites have been reported as pests of pepino plants worldwide (Table 4) (Galbreath and Clearwater 1983, Larraín 2002, Akyazi 2012). Among these pests, 16 species are distributed in Japan, but only three species, *Trichoplusia ni*, *Polyphagotarsonemus latus*, and *Tetranychus urticae*, were evaluated as pests of pepino in the present study. This indicates that the remaining 13 species are very likely to be potential pests on pepino in Japan. Therefore, at least 26 insect and mite species, including the 13 currently known and the 13 potential ones in Japan, will be recognized as pests of pepino in the near future if the cultivation of pepinos spreads throughout the Japanese Archipelago.

The presence of pests can directly affect agricultural production and it may contribute to the transmission of plant viruses followed by economic losses. *Bemisia tabaci*, well-known as one of the most important whiteflies in terms of virus transmission, is widely distributed in the world, and is a vector of viruses of the genera *Begomovirus*, *Carlavirus*, *Crinivirus*, *Ipomovirus*, and *Torradovirus* (Navas-Castillo et al. 2011). Of these transmissible viruses, *Tomato yellow leaf curl virus* (TYLCV; *Begomovirus*) is the most devastating causal virus on tomato crops in many tropical, subtropical and temperate regions worldwide (Moriones and Navas-Castillo 2000). In Japan, TYLCV spread along with whitefly and indicated its dispersion throughout 38 prefectures by 2014 since the occurrence of TYLCV on tomato was first reported in Japan (Kato et al. 1998, Matsuura and Hoshino 2008, Ohnishi et al. 2016). However, there is no report of TYLCV incidence on pepino plants to date. Pepino can be regarded as a potential host for TYLCV through host adaptation or mutant as long as TYLCV-acquired vectors are present. Therefore, continuous monitoring of the distribution of both TYLCV and its vector is required. Some other species of insects and mites found on pepino plants in our research fields are also regarded as virus vectors (Table 5). Although some transmitted viruses cannot infect pepino plants, they may provide a habitat for virus vectors, which may impact crop ecosystems as well as virus-vector systems.

We found virus-like symptoms, showing mottle and deformation on pepino leaves. However, none of the tested viruses were detected in any plant. Our internet-based image searching results showed that the symptoms were similar to those on pepper or chili plants by *Polyphagotarsonemus latus*. Grinberg et al. (2005) described that young leaves were usually affected by *P. latus* and consequently showed distortion and leaf-curl downwards. This suggests that our symptomatic leaves might be caused by *P. latus*. Further research is required to reveal any effect of *P. latus* on pepino.

No related virus was detected in the present study, whereas two virus species had been detected from pepino plants in Japan: *Alfalfa mosaic virus* (AMV) and *Cucumber mosaic virus* (CMV) inducing chlorotic ring spots and mosaic symptoms on pepino plants, respectively (Honda et al. 1986). Moreover, *Pepino mosaic virus* (PepMV) was first isolated from pepinos showing yellow mosaic symptoms in Peru in 1974 (Jones et al. 1980). Currently, PepMV has become a major pathogen of tomato plants worldwide and is one of the quarantine pathogens strictly prohibited from entry into Japan. Pepino latent virus (PepLV; later reclassified as *Potato virus S* (PVS)) was detected from pepino cuttings in New Zealand, even though pepino plants with no symptoms had been imported from Chile (Thomas et al. 1980). With the increase in pepino production in China, *Potato virus H* (PVH) infected pepino, with no obvious symptoms (Abouelnasr et al. 2014) (Table 6). Pests and pathogens in many crops cause tremendous losses both in terms of quantity and quality. Monitoring, detection, and identification of pests and pathogens prior to the introduction of a new crop or during production are important to efficiently regulate their future damage. Our findings will aid in understanding the incidence of pests and viral diseases on pepino plants and developing better crop production systems to combat pests and diseases.

## Supplementary Material

XML Treatment for Insecta

XML Treatment for Thysanoptera

XML Treatment for Thripidae

XML Treatment for Frankliniella intonsa

XML Treatment for Hemiptera

XML Treatment for Aleyrodidae

XML Treatment for Bemisia tabaci

XML Treatment for Aphididae

XML Treatment for Aphis gossypii

XML Treatment for Pseudococcidae

XML Treatment for Phenacoccus solani

XML Treatment for Miridae

XML Treatment for Campylomma livida

XML Treatment for Coleoptera

XML Treatment for Chrysomelidae

XML Treatment for Epitrix hirtipennis

XML Treatment for Lepidoptera

XML Treatment for Noctuidae

XML Treatment for Spodoptera litura

XML Treatment for Trichoplusia ni

XML Treatment for Diptera

XML Treatment for Agromyzidae

XML Treatment for Liriomyza sativae

XML Treatment for Arachnida

XML Treatment for Acari

XML Treatment for Tarsonemidae

XML Treatment for Polyphagotarsonemus latus

XML Treatment for Tetranychidae

XML Treatment for Tetranychus urticae

## Figures and Tables

**Table 1.** Primers used for virus detection in this study.

Target virus	Primer	Strand	Sequence (5' to 3')	Expected amplicon size (bp)	Reference
*Alfalfa mosaic virus* (AMV)	AMV-F2	+	ATCATGAGTTCTTCACAAAAGAA	670	Xu and Nie (2006)
	AMV-R2	-	TCAATGACGATCAAGATCGTC		
*Cucumber mosaic virus* (CMV)	CPTALL-5	+	YASYTTTDRGGTTCAATTCC	950	Choi et al. (1999)
	CPTALL-3	-	GACTGACCATTTTAGCCG		
Genus *Carlavirus*	Carla-uni	+	GGAGTAACCGAGGTGATACC	120	Badge et al. (1996) modified
	oligo dT	-	T_18_		
Genus *Potexvirus*	Potex 5	+	CAYCARCARGCMAARGAYGA	600	van der Vlugt and Berendsen (2002)
	Potex 2RC	-	AGCATRGCNSCRTCYTG		
Genus *Potyvirus*	CIFor	+	GGIVVIGTIGGIWSIGGIAARTCIAC	700	Ha et al. (2008)
	CIRev	-	ACICCRTTYTCDATDATRTTIGTIGC		

**Table 2.** List of insect and mite pests found on pepino plants in the Atsugi Campus of Tokyo University of Agriculture (TUA), Kanagawa, Japan.

Class	Order	Family	Species	Suvery plots
Insecta	Thysanoptera	Thripidae	*Frankliniella intonsa* (Trybom, 1895)	A C
Insecta	Hemiptera	Aleyrodidae	*Bemisia tabaci* (Gennadius, 1889)	B C
Insecta	Hemiptera	Aphididae	*Aphis gossypii* Glover, 1877	A C
Insecta	Hemiptera	Pseudococcidae	*Phenacoccus solani* Ferris, 1918	C
Insecta	Hemiptera	Miridae	*Campylomma livida* Reuter, 1885	A
Insecta	Coleoptera	Chrysomelidae	*Epitrix hirtipennis* (Melsheimer, 1847)	A C
Insecta	Diptera	Agromyzidae	*Liriomyza sativae* Blanchard, 1938	C
Insecta	Lepidoptera	Noctuidae	*Spodoptera litura* (Fabricius, 1775)	A B
Insecta	Lepidoptera	Noctuidae	*Trichoplusia ni* (Hübner, 1803)	B
Arachnida	Acari	Tarsonemidae	*Polyphagotarsonemus latus* (Banks, 1904)	C
Arachnida	Acari	Tetranychidae	*Tetranychus urticae* Koch, 1836	A C

**Table 3.** Insect and mite pests of pepino plants previously recorded in Japan.

Class	Order	Family	Group (of species and its allies)	Species	References
Insecta	Hemiptera	Aleyrodidae	-	*Trialeurodes vaporariorum* (Westwood, 1856)	Furusato (1984), Takahashi (1985), Takagi (1985), Kita (1986), Odagiri et al. (1986), Ozawa (1986)
Insecta	Hemiptera		aphids	-	Takagi (1985), Takahashi (1985), Kita (1986), Odagiri et al. (1986), Ozawa (1986), Takahashi (1986)
Insecta	Lepidoptera	Noctuidae	*Helicoverpa* spp.	-	Odagiri et al. (1986)
Insecta	Lepidoptera	Noctuidae	*Spodoptera* spp.	-	Odagiri et al. (1986)
Insecta	Lepidoptera	Gelechiidae	-	*Phthorimaea operculella* (Zeller, 1873)	Ozawa (1986)
Insecta	Lepidoptera	unspecified	green caterpillars	-	Furusato (1986)
Arachnida	Acari	unspecified	mites	-	Furusato (1984), Kita (1986), Odagiri et al. (1986)
Arachnida	Acari	Tetranychidae	spider mites	-	Takahashi (1985), Takagi (1985), Ozawa (1986), Takahashi (1986)
Arachnida	Acari	Tetranychidae	-	*Tetranychus urticae* Koch, 1836	Ozawa (1986)

**Table 4.** Insect and mite pests of pepino plants previously recorded worldwide (excluding Japan).

Class	Order	Family	Species	Country recorded as a pest	References	Notes
Insecta	Orthoptera	Acrididae	*Schistocerca cancellata* (Serville, 1838)	Chili	Larraín (2002)	
Insecta	Thysanoptera	Thripidae	*Frankliniella occidentalis* (Pergande, 1895)	Chili	Larraín (2002)	Distributed in Japan
Insecta	Thysanoptera	Thripidae	*Thrips tabaci* Lindeman, 1889	Chili	Larraín (2002)	Distributed in Japan
Insecta	Hemiptera	Aphididae	*Aulacorthum solani* (Kaltenbach, 1843)	Chili	Larraín (2002)	Distributed in Japan
Insecta	Hemiptera	Aphididae	*Macrosiphum euphorbiae* (Thomas, 1878)	Chili	Larraín (2002)	Distributed in Japan
Insecta	Hemiptera	Aphididae	*Myzus persicae* (Sulzer, 1776)	Chili	Larraín (2002)	Distributed in Japan
Insecta	Hemiptera	Pseudococcidae	*Phenacoccus solenopsis* Tinsley, 1898	Chili	Larraín (2002)	Distributed in Japan
Insecta	Hemiptera	Pseudococcidae	*Pseudococcus viburni* (Signoret, 1875)	Chili	Larraín (2002)	Distributed in Japan
Insecta	Hemiptera	Psyllidae	*Russelliana solanicola* Tuthill, 1959	Chili	Larraín (2002)	
Insecta	Hemiptera	Triozidae	*Trioza chenopodii* Reuter, 1876	Chili	Larraín (2002)	Distributed in Japan
Insecta	Hemiptera	Cicadellidae	*Xerophloea viridis* (Fabricius, 1794)	Chili	Larraín (2002)	
Insecta	Hemiptera	Cicadellidae	*Paratanus exitiosus* (Beamer, 1943)	Chili	Larraín (2002)	
Insecta	Diptera	Agromyzidae	*Liriomyza huidobrensis* (Blanchard, 1926)	Chili	Larraín (2002)	Distributed in Japan
Insecta	Diptera	Tephritidae	*Rhagoletis nova* (Schiner, 1868)	Chili	Larraín (2002)	
Insecta	Lepidoptera	Noctuidae	*Agrotis bilitura* Guenée, 1852	Chili	Larraín (2002)	
Insecta	Lepidoptera	Noctuidae	*Copitarsia turbata* (Herrich-Schaeffer, 1855)	Chili	Larraín (2002)	
Insecta	Lepidoptera	Noctuidae	*Trichoplusia ni* (Hübner, [1803])	Chili	Larraín (2002)	Recognized as a pest in Japan by this study
Insecta	Lepidoptera	Sphingidae	*Manduca sexta* (Linnaeus, 1763)	Chili	Larraín (2002)	Distributed in Japan
Insecta	Lepidoptera	Gelechiidae	*Phthorimaea operculella* (Zeller, 1873)	Chili	Larraín (2002)	Distributed in Japan
Insecta	Lepidoptera	Gelechiidae	*Symmetrischema tangolias* (Gyen, 1913)	Chili	Larraín (2002)	
Insecta	Lepidoptera	Gelechiidae	*Tuta absoluta* (Meyrick, 1917)	Chili	Larraín (2002)	Distributed in Japan
Insecta	Lepidoptera	Crambidae	*Sceliodes cordalis* (Doubleday, 1843)	New Zealand	Galbreath and Clearwater (1983)	
Arachnida	Acari	Eriophyidae	*Aculops lycopersici* (Tryon, 1917)	Chili, Turkey	Larraín (2002), Akyazi (2012)	Distributed in Japan
Arachnida	Acari	Tarsonemidae	*Polyphagotarsonemus latus* (Banks, 1904)	Chili	Larraín (2002)	Recognized as a pest in Japan by this study
Arachnida	Acari	Tetranychidae	*Tetranychus urticae* Koch, 1836	Chili	Larraín (2002)	Recognized as a pest in Japan by this study

**Table 5.** Insects or mites of pepino plants involved in virus transmission

Insect vector	Transmissible genus or species of virus	Reference
*Frankliniella intonsa*	*Groundnut ringspot virus*	Riley et al. (2011)
	*Impatiens necrotic spot virus*	
	*Tomato chlorotic spot virus*	
	*Tomato spotted wilt virus*	
*Bemisia tabaci*	Genus *Begomovirus*	Navas-Castillo et al. (2011)
	Genus *Carlavirus*	
	Genus *Crinivirus*	
	Genus *Ipomovirus*	
	Genus *Torradovirus*	
*Aphis gossypii*	*Cucumber mosaic virus* (CMV)	Pinto et al. (2008)
	*Papaya ringspot virus* (PRSV)	
	*Tobacco ringspot virus* (TRSV)	Stace-Smith (1985)
	*Zucchini yellow mosaic virus* (ZYMV)	Pinto et al. (2008)
*Epitrix hirtipennis*	*Tobacco ringspot virus* (TRSV)	Stace-Smith (1985)
*Liriomyza sativae*	*Celery mosaic virus* (CeMV)	Zitter and Tsai (1977)
	*Watermelon mosaic virus* strain 1*^1^	
	*Watermelon mosaic virus* strain 2	
*Tetranychus urticae*	*Tobacco mosaic virus* (TMV)*^2^	Orlob (1968)
	*Potato virus X* (PVX)*^2^	

**Table 6.** Viruses infecting pepino plants naturally around the world.

Family	Genus	Species	Acronym	Symptoms on pepino	First reported country	Reference
* Alphaflexiviridae *	* Potexvirus *	*Pepino mosaic virus*	PepMV	yellow mosaic in young leaves	Peru	Jones et al. (1980)
* Betaflexiviridae *	* Carlavirus *	Potato virus H	PVH	symptomless	China	Abouelnasr et al. (2014)
		Pepino latent virus*^3^	PepLV	symptomless	New Zealand	Thomas et al. (1980)
* Bromoviridae *	* Alfamovirus *	*Alfalfa mosaic virus*	AMV	chlorotic ring spot	Japan	Honda et al. (1986)
	* Cucumovirus *	*Cucumber mosaic virus*	CMV	mosaic	Japan	Honda et al. (1986)

## Footnotes

*1 Reclassified as PRSV (Purcifull et al. 1984)

*2 Experimentally transmissible

*3 Reclassified as an isolate of *Potato virus S* (PVS) based on biological and serological characteristics (Dolby and Jones 1988).
